# Leveraging dysregulated tumor metabolism for targeting anticancer bacteria

**DOI:** 10.1126/sciadv.ads1630

**Published:** 2025-06-13

**Authors:** Akeem Santos, Zeneng Wang, Rashmi Bharti, Goutam Dey, Naseer Sangwan, William Baldwin, Ajay Zalavadia, Alex Myers, Olivia G. Huffman, Justin D. Lathia, Stanley L. Hazen, Ofer Reizes, Mohammed Dwidar

**Affiliations:** ^1^ Department of Cardiovascular & Metabolic Sciences, Lerner Research Institute, Cleveland Clinic Foundation, Cleveland, OH, USA.; ^2^ Center for Microbiome and Human Health, Lerner Research Institute, Cleveland Clinic Foundation, Cleveland, OH, USA.; ^3^ Cleveland Clinic Lerner College of Medicine, Case Western Reserve University, Cleveland, OH, USA.; ^4^ Department of Inflammation & Immunity, Lerner Research Institute, Cleveland Clinic Foundation, Cleveland, OH, USA.; ^5^ Imaging Core, Lerner Research Institute, Cleveland Clinic Foundation, Cleveland, OH, USA.; ^6^ Case Comprehensive Cancer Center, Cleveland, OH, USA.; ^7^ Rose Ella Burkhardt Brain Tumor & Neuro-Oncology Center, Cleveland Clinic Foundation, Cleveland, OH, USA.; ^8^ Department of Cardiovascular Medicine, Heart, Vascular and Thoracic Institute, Cleveland Clinic Foundation, Cleveland, OH, USA.

## Abstract

Widespread application of bacterial-based cancer therapy is limited because of the need to increase therapeutic bacteria specificity to the tumor to improve treatment safety and efficacy. Here, we harness the altered tumor metabolism and specifically elevated kynurenine accumulation to target engineered bacteria to the cancer site. We cloned and leveraged kynurenine-responsive transcriptional regulator (KynR) with its cognate promoter in *Escherichia coli*. Optimizing KynR expression coupled with overexpressing kynurenine transporter and amplifying the response through plasmid copy number–based signal amplification enabled the response to kynurenine at the low micromolar levels. Knocking out genes essential for cell wall synthesis and supplying these genes via kynurenine-controlled circuits allowed tuning *Salmonella enterica* growth in response to kynurenine. Our kynurenine-controlled *S. enterica* (hereafter named AD95+) showed superior tumor specificity in breast and ovarian cancer murine models compared to *S. enterica* VNP20009, one of the best characterized tumor-specific strains. Last, AD95+ showed anticancer properties compared to vehicle controls, demonstrating the potential as an anticancer therapeutic.

## Teaser

Metabolic sensors selectively home anticancer bacteria to solid tumors.

## INTRODUCTION

Using bacteria as cancer therapeutics offers a promising treatment option for solid tumor cases where standard-of-care approaches are inadequate and ineffective. This strategy is based on the natural tendency of some bacterial strains such as *Salmonella enterica*, *Escherichia coli*, and *Clostridium novyi* to preferentially grow within the tumor compared to normal tissues. Bacterial growth causes both a direct toxic effect on the tumor cells and activation of the immune system, leading to enhanced antitumor immunity (*1*–*3*). Bacterial tumor tropism has been attributed to multiple factors including nutrient availability, protection from immune surveillance, and the hypoxic environment within solid tumors in the case of anaerobic bacteria (*4*). With the latest advances in bacterial synthetic biology, bacterial-based cancer therapies are moving from concept to potential clinical application. Several clinical trials are ongoing using genetically modified bacterial strains as either monotherapy or in combination with other therapies including immune checkpoint inhibitors (e.g., NCT05038150, NCT00004988, NCT01675765, NCT01924689, NCT03435952, etc.). Furthermore, some bacterial-based cancer therapies have demonstrated clinical success and are routinely used in cancers [e.g., Bacillus Calmette-Guerin therapy for bladder cancer (*5*)]. However, a major limitation for widespread application of bacterial-based cancer therapies is the need to improve the specificity of the bacteria to the cancer site, especially if these bacteria are engineered to carry a cytotoxic payload (*4*).

For *S. enterica*, one of the most promising and widely tested bacteria as cancer therapeutics, several previous approaches focused on attenuating its known toxicity and improve its tumor targeting (*6*–*8*). Of note, the *S. enterica* VNP20009 strain was engineered with mutations in *purI* and *msbB* genes, making it auxotrophic to alanine and less stimulatory to tumor necrosis factor–α (TNFα) production (*9*). This strain was among the most promising when tested in murine models in terms of specificity to the tumor (*10*). Initial attempts testing VNP20009 in clinical trials found that it was ineffective in causing tumor regression at the used doses and was associated with toxicity limitations at higher doses (*11*). This highlights the need to find alternative methods to engineer *S. enterica* to home specifically to tumors and enhance the safety profile without reducing efficacy. We hypothesize that this can be achieved through controlling *S. enterica* gene expression and growth in response to specific metabolites within the tumor microenvironment.

Kynurenine is a catabolite of tryptophan, which is overproduced in solid tumors (*12*–*24*). Kynurenine overproduction serves as one of several mechanisms tumors use to escape immune surveillance (*25*, *26*). Kynurenine overproduction is caused by enhanced expression of IDO1 (indoleamine 2,3-dioxygenase 1) enzyme (the first and rate-limiting step in the kynurenine pathway) with some studies suggesting a role for its isozyme TDO2 (tryptophan 2,3-dioxygenase), which catalyzes the same reaction (*27*, *28*). In most forms of human cancers, high IDO1 expression is positively correlated with poor prognosis (*19*). As such, several IDO1 inhibitors have entered different phases of oncology clinical trials (*28*, *29*). The level of IDO1 expression, and hence, the increase in kynurenine production, differs between tumors and is dependent on T cell infiltration with associated inflammatory cytokines, especially interferon-γ (IFN-γ) (*30*).

In this study, we aim to harness this local accumulation of the immunosuppressive kynurenine in solid tumors as a cue to target bacterial-based therapeutics to solid tumors through engineering kynurenine-responsive bacterial genetic circuits and use these circuits to control gene expression and growth of the anticancer bacteria. While our approaches can be applied to a variety of solid tumors, we focus here on ovarian cancer (OC) and triple negative breast cancer (TNBC) tumor models. These two cancers have different microenvironments with TNBC developing within the mammary glands, while OC is initiated in the fallopian tube and disseminates throughout the peritoneal cavity. Breast cancer is one of the most common cancers in women worldwide and a leading cause for cancer-related death. Among the different breast cancer subtypes, TNBC accounts for ~15% of all breast cancer cases (*31*). TNBC is estrogen receptor negative, progesterone receptor negative, and human epidermal growth factor receptor 2 negative. This subtype is considered the most aggressive, least responsive for treatment and usually has the worst prognosis (*31*–*33*). Likewise, OC is the deadliest gynecological cancer. A woman’s risk of getting OC during her lifetime is about 1 in 78 (*34*). There is an urgent need to find alternative treatment options for both cancers with current average 5-year survival rates of 50% for OC (*34*, *35*) and 77% for TNBC (*36*). Previous studies found that the kynurenine pathway is elevated in both cancers and contributes to immunosuppression (*12*–*19*), providing a rationale for using kynurenine as a signal to target engineered bacteria to both cancers.

## RESULTS

### Kynurenine is enriched in malignant tumors compared to other tissues in both human and preclinical murine models for TNBC and OC

The ability to target bacteria to tumors requires controlling the growth of these bacteria in response to a tumor-specific signal. We use kynurenine as it is a small secreted molecule enriched in solid tumors. To first confirm whether the kynurenine pathway is elevated in TNBC, we examined the changes in the expression levels of the key kynurenine-producing enzymes, IDO1, and TDO2 in TNBC using the publicly available transcriptomics data in The Cancer Genome Atlas (www.cancer.gov/tcga). The analysis reveals that IDO1 expression is significantly higher in TNBC compared to healthy breast tissue samples (fig. S1A). Likewise, TDO2 expression was also elevated (fig. S1B).

**Fig. 1.**
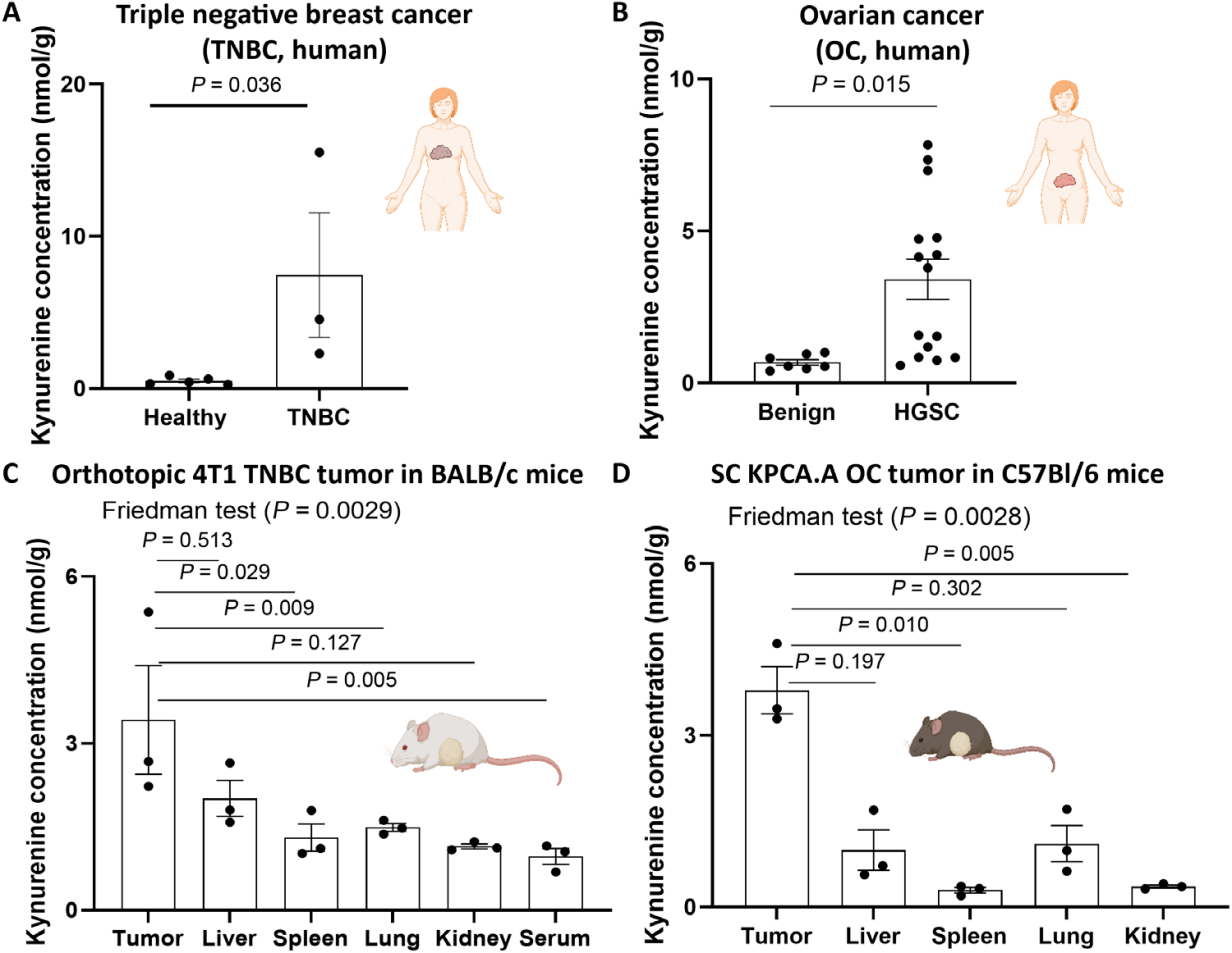
Kynurenine is overproduced in TNBC and OC tumors. (**A**) Kynurenine level in patient TNBC versus healthy breast tissue specimens (*n* = 5 and 3 for healthy and TNBC tissue specimens, respectively). (**B**) Kynurenine level in patient high-grade serous OC (HGSOC) versus benign ovarian specimens (*n* = 7 and 15 for benign ovarian and HGSOC specimens, respectively). (**C**) Syngeneic orthotopic 4T1 TNBC tumors were developed in the mammary fat pads of BALB/c mice. Tissues were harvested when tumors were ~200 mm^3^. Kynurenine levels in the different tissues were measured through LC-MS/MS (*n* = 3). (**D**) C57Bl/6J mice were injected subcutaneously with KPCA.A murine OC cells. Tissues were harvested when tumor diameters were ~1600 mm^3^ (*n* = 3). All samples were homogenized, diluted, and analyzed through LC-MS/MS. Bars and lines show the means ± SE. Significance was tested using Mann-Whitney test for (A) and (B) and using Friedman test for (C) and (D). Created in BioRender. Dwidar, M. (2025) https://BioRender.com/d55t198.

We then looked at the kynurenine concentrations in malignant tissues compared to their healthy counterparts. We performed liquid chromatography–tandem mass spectrometry (LC-MS/MS) analyses on a total of eight independent patient breast specimens [three TNBC and five control (healthy)]. Kynurenine levels in TNBC tissue specimens were 16-fold higher compared to the levels in healthy breast tissue (TNBC mean tissue content, 7.5 ± 4.1 nmol/g; *n* = 3; healthy tissue mean concentration, 0.5 ± 0.1 nmol/g; *n* = 5; *P* = 0.036) (Fig. 1A). Likewise, we analyzed a total of 22 patient ovarian tumor specimens [7 benign and 15 high grade serous OC (HGSOC)] from human subjects. We found that mean kynurenine content in malignant tumor specimens is significantly higher compared to benign tumor specimens (3.4 ± 1.2 nmol/g versus 0.7 ± 0.1 nmol/g for malignant and benign tumors, respectively, *P* = 0.01; Fig. 1B).

For the bacteria to home to tumor kynurenine, the concentration in tumors should be higher compared to those in other organs. To test for this hypothesis, we injected the syngeneic TNBC cell line 4T1 (*37*, *38*) orthotopically in the mammary fat pad and the HGSOC murine cell line KPCA.A (*39*) subcutaneously in BALB/c and C57BL/6 mice. At the endpoint, we harvested multiple tissues and analyzed kynurenine content. Among all tissues examined, kynurenine was the highest in tumors with averages of 3.4 ± 1.2 and 3.8 ± 0.4 nmol/g compared to averages of 1.4 ± 0.1 and 0.7 ± 0.1 nmol/g in all other tissues, respectively (Fig. 1, C and D). Collectively, these results indicated that to use kynurenine as a tumor signal to target therapeutic bacteria, we need to engineer a kynurenine-sensing system in bacteria capable of responding to kynurenine at the low micromolar range (1 to 10 μM) with high ON/OFF ratio.

### Leveraging the performance of a natural kynurenine-sensing system through plasmid copy number–based signal amplification

In the prokaryotic world, some bacterial species such as *Pseudomonas aeruginosa*, *Burkholderia cepacia*, and others have an aerobic kynurenine catabolic pathway for tryptophan similar to the metabolic pathway in eukaryotes (*40*–*43*). Bacterial kynurenine is produced as an intermediate metabolite before it is further catabolized into anthranillic acid or other metabolites. This kynurenine pathway is regulated through the kynurenine-responsive transcriptional regulator (KynR) (*40*, *44*). KynR binds to kynurenine, leading to transcriptional activation of downstream enzymes in the pathway (fig. S2A).

**Fig. 2.**
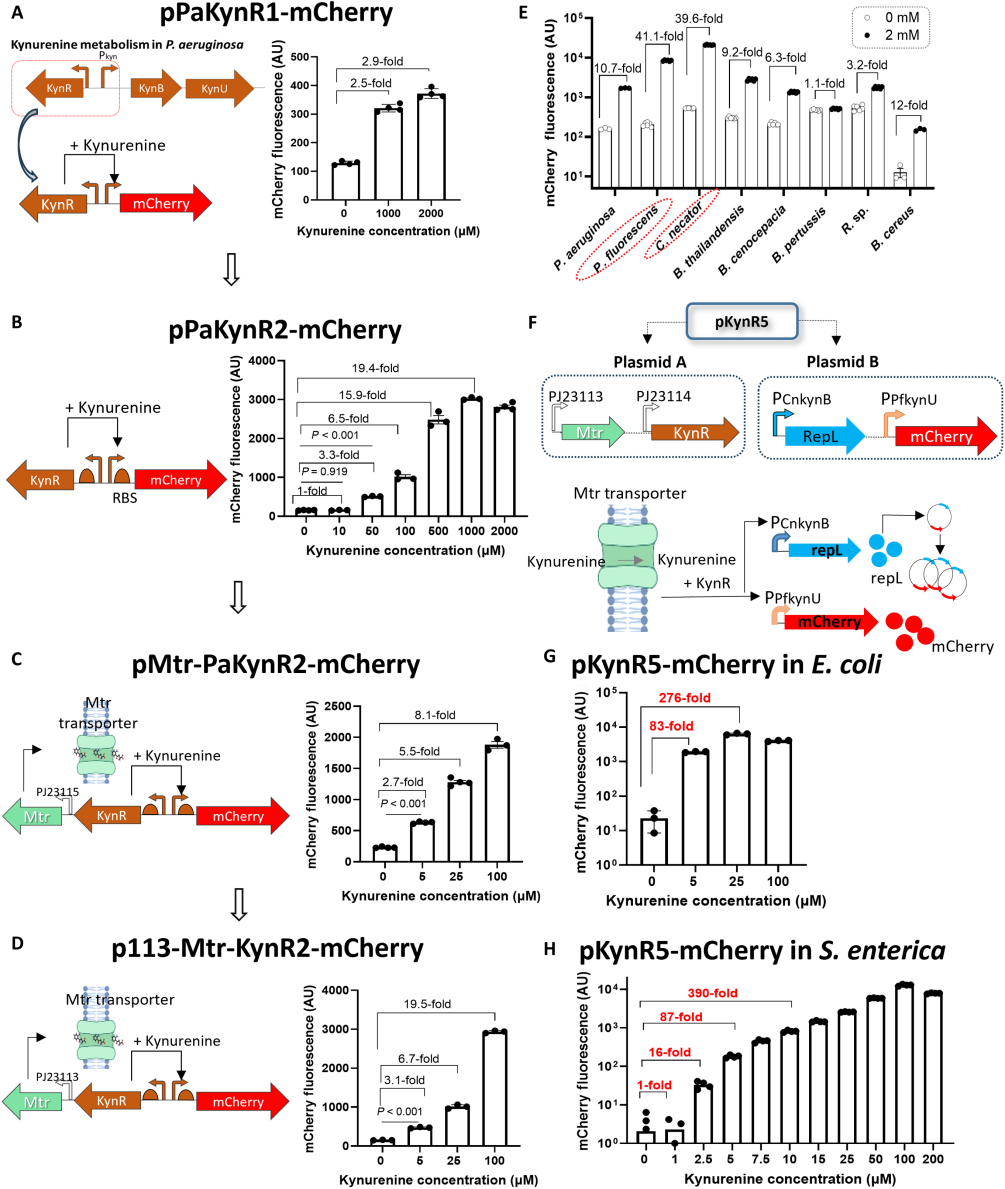
A KynR regulator with its cognate promoter (P_kyn_) was cloned on a plasmid system and optimized in *E. coli* and then transferred to *S. enterica.* (**A**) The original construct pPaKynR1-mCherry, in which KynR with its cognate promoter (P_kynB_) was cloned from *P. aeruginosa* upstream of the mCherry reporter on a plasmid, demonstrated only modest performance in *E. coli* K12. AU, arbitrary units. (**B**) Further optimization through modifying the RBS sequence for *kynR* and *mCherry* genes resulted in pPaKynR2-mCherry. (**C**) The Mtr transporter was overexpressed on the same plasmid to make the pMtr-PaKynR2-mCherry system. (**D**) Expression of the cloned Mtr transporter was tuned to further enhance the sensitivity to kynurenine in p113-Mtr-PaKynR2-mCherry. (**E**) Comparing P_kyn_ promoter sequences from different bacterial species for their performance in *E. coli.* Except for *B. cereus* P_kynU_, all promoter sequences were tested on a plasmid that harbors *P. aeruginosa* KynR. (**F**) Schematic diagram showing the optimized two-plasmid system (pKynR5-mCherry). pKynR5-mCherry used the *kynR* gene from *P. aeruginosa* together with the *mtr* transporter on plasmid A. The kynurenine-inducible promoters P_CnkynB_ and P_PfkynU_ from *P. fluorescens* and *C. nectar*, respectively, were placed on plasmid B upstream of the plasmid replication gene *repL* and the reporter *mCherry*, respectively. (**G** and **H**) Performance of the optimized dual-plasmid system, pKynR5-mCherry, in *E. coli* and *S. enterica*, respectively. The recombinant *E. coli* (A to G) or *S. enterica* (H) harboring each of these plasmids were cultured in M9 media supplemented with 0.4% glucose +/− kynurenine (*n* = 3 to 6), and mCherry fluorescence/OD_600_ was measured. Plotted are the means ± SE. Created in BioRender. Dwidar, M. (2025) https://BioRender.com/d55t198.

In our initial experiments, we cloned the *kynR* gene together with its cognate promoter (P_kynB_) from *P. aeruginosa* in front of the *mCherry* gene on a plasmid (pPakynR1-mCherry). Transforming this plasmid into *E. coli* K12 resulted in a modest response to kynurenine (2.5- and 3-fold induction when kynurenine was added to the culture medium at concentrations of 1 and 2 mM, respectively) (Fig. 2A). Optimizing *kynR* expression and the ribosomal binding site (RBS) sequence of mCherry resulted in pPaKynR2-mCherry version, which showed a better response to kynurenine (3.3-fold induction at a 50 μM kynurenine concentration) (Fig. 2B). We then aimed at further improving the response through overexpressing a transporter to allow for enhanced import of kynurenine. *E. coli* has three different aromatic amino acid transporters, which could serve this function (namely, Mtr, AroP, and TnaB) (*45*). Expressing each of these three transporters in the pPaKynR2-mCherry plasmid showed that they all enhance the response to kynurenine, albeit at different degrees with the highest response found for Mtr overexpression (Fig. 2C and fig. S3). Subsequently, we reduced Mtr transporter expression by replacing the upstream medium-strength PJ23115 promoter (https://parts.igem.org/Part:BBa_J23115) with the weaker PJ23113 promoter. This resulted in p113-Mtr-paKynR2-mCherry version, which demonstrated an induction ratio of 3.1-fold at a kynurenine concentration of 5 μM (Fig. 2D). In parallel, we tested other kynurenine-responsive promoter sequences from different bacterial strains. *Pseudomonas fluorescens* P_kynU_ and *Cupriavidus necator* P_kynB_ sequences demonstrated improved performance (higher ON/OFF ratio) compared to the P_kynB_ sequence cloned from *P. aeruginosa* (Fig. 2E and fig. S2, B to D).

To maximize the ON/OFF switching ratio of the gene circuit, we adapted a technique to allow kynurenine to not only induce *mCherry* transcription but also induce amplification of the *mCherry* gene copy number. To achieve this goal, we chose the two promoter sequences *P. fluorescens* P_kynU_ and *C. necator* P_kynB_ because these two promoters showed the highest induction ratios in the presence of kynurenine (Fig. 2E). For this system, which we called pKynR5-mCherry, the genes for the transcriptional regulator *P. aeruginosa* KynR, together with the Mtr transporter, were placed on one plasmid (plasmid A) downstream of PJ23114 and PJ23113 synthetic promoters, respectively. The reporter of interest (*mCherry*) was placed on a different plasmid (plasmid B). Plasmid B has two origins of replication (*46*, *47*): mini-F origin for stable plasmid maintenance and P1 phage–derived origin (*oriL*), which is located within the coding region of the replication protein RepL (*48*). The *P. fluorescens* P_kynU_ promoter was placed upstream of the reporter gene of interest (*mCherry*), while the *C. necator* P_kynB_ promoter was placed upstream of the plasmid B replication gene (*repL*) (Fig. 2F). When kynurenine is present in the medium, it is actively taken into the bacterial cell through the Mtr transporter expressed from plasmid A*.* Once inside the bacterial cell, kynurenine binds to KynR expressed from plasmid A and activates the transcription of both mCherry and the replication protein (RepL) of plasmid B. RepL expression facilitates plasmid B replication, resulting in an increased copy number of the reporter gene (*mCherry*), which in turn boosts mCherry protein expression. Using this system, we reached an ON/OFF ratio of 83- and 87-fold at kynurenine concentrations of 5 μM when initially tested in *E. coli* and when moved to *S. enterica*, respectively (Fig. 2, G and H). This system shows a sharp (exponential) response in the 1 to 10 μM range [the typical concentration in tumors based on the literature (*20*, *49*) and as shown in Fig. 1]. Similar results were also found in both *E. coli* and *S. enterica* when mCherry was replaced by the nLuc reporter (fig. S4).

Because Mtr serves as a tryptophan transporter, we tested how the pKynR5-mCherry system behaves in the presence of tryptophan. As expected, the results indicated that the pKynR5-mCherry response to kynurenine is reduced proportionally as the tryptophan concentration in the media is increased (fig. S5). However, even at a tryptophan concentration of 50 μM, the pKynR5-mCherry system still showed a high ON/OFF ratio (16.3-fold) at a 5 μM kynurenine concentration.

### Engineered *S. enterica* responds to kynurenine secreted by murine OC and TNBC cell lines

**Fig. 3.**
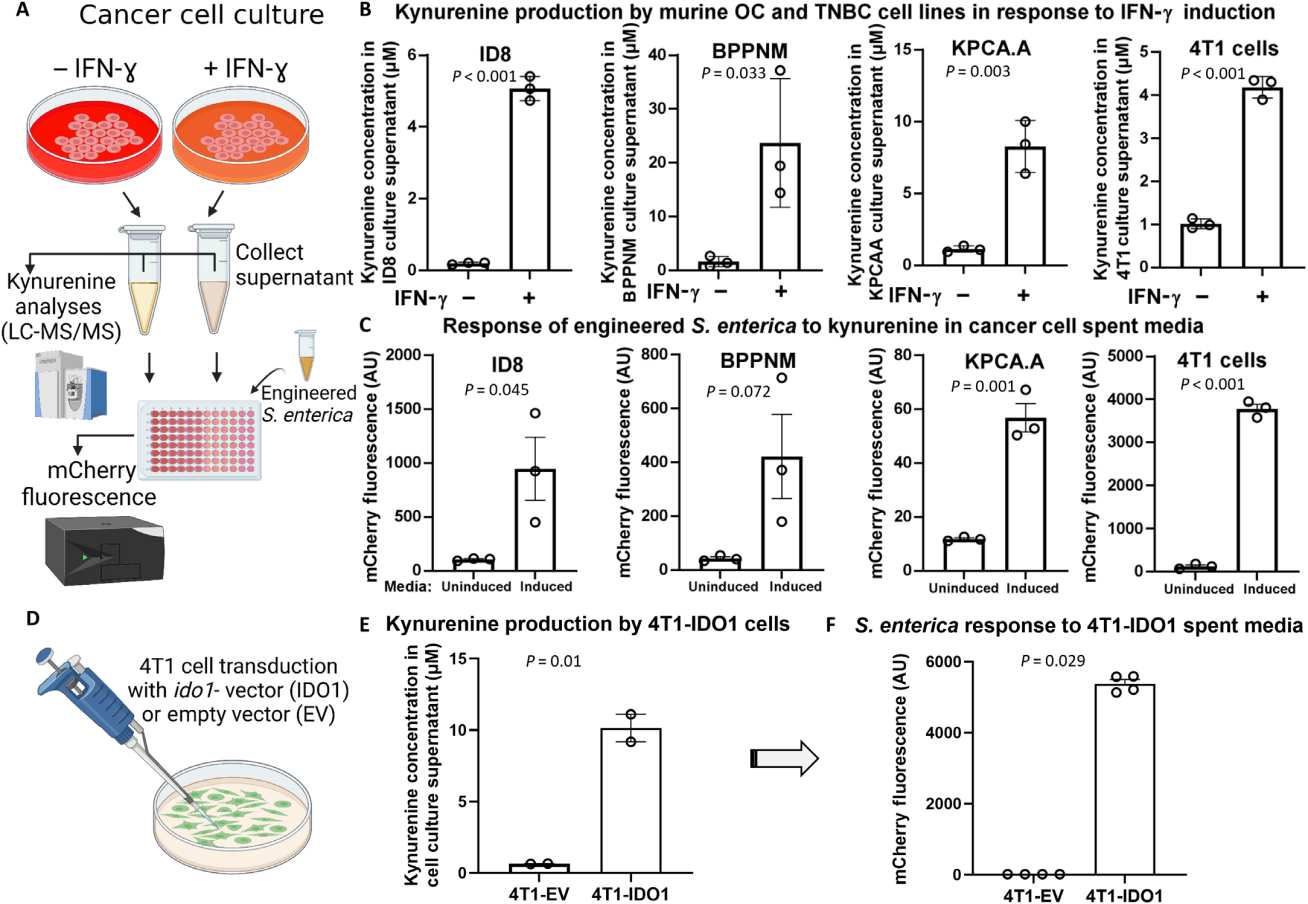
Engineered *S. enterica* responds to kynurenine secreted in cancer cell spent media. (**A**) Schematic diagram for the experiment. (**B**) Murine cancer cell lines (100,000 cells) were cultured in DMEM-serum media +/− IFN-γ (200 ng/ml). Spent media were analyzed for kynurenine after 72 hours. (**C**) Spent media of IFN-γ–induced/uninduced cancer cells were aliquoted in a 96-well plate. *S. enterica*/pkynR5-mCherry was then added to each well. Bacterial growth and mCherry fluorescence were monitored. (**D**) 4T1-Luc cancer cells were transduced with either IDO1-exressing vector (IDO1) or control empty vector (EV). (**E** and **F**) Both cell lines were cultured, and spent media were analyzed for kynurenine and used for culturing engineered *S. enterica*/pkynR5-mCherry. *n* = 2 to 4 for each. Plotted are the means ± SE. Created in BioRender. Dwidar, M. (2025) https://BioRender.com/d55t198.

To first assess kynurenine production by cancer cells, OC and TNBC cells were cultured in tissue culture dishes for 72 hours, with or without IFN-γ, the primary inducer of the IDO1 enzyme and kynurenine production in tumors. The kynurenine concentration in the culture supernatant was analyzed using LC-MS/MS (Fig. 3A). The results demonstrated that IFN-γ addition induced kynurenine production for each of the tested cell lines, albeit to different extents depending on the cell line (Fig. 3B). This included the widely used OC ID8 cell line (*50*); the recently developed KPCA.A and BPPNM, which both are genetically defined and establish intraperitoneal tumors recapitulating high-grade serous OC (HGSOC) (*39*); and the murine TNBC cell line 4T1 (*37*, *38*). We then cultured the engineered *S. enterica* harboring pkynR5-mCherry using spent media from each cancer cell line. The results showed that engineered *S. enterica* responded to kynurenine in the spent media as expected, reflecting the kynurenine concentrations in each sample (Fig. 3C).

To further validate our kynurenine-responsive genetic circuit, we transduced 4T1 cancer cells with either an *ido1*-expressing lentiviral vector or an empty vector, creating the 4T1-IDO1 and 4T1-EV cell lines, respectively. Analyzing the culture spent media of both cell lines confirmed the constitutive (without IFN-γ induction) production of kynurenine by 4T1-IDO1 cells (Fig. 3, D and E). When spent media from both cell lines were used to culture *S. enterica*/pkynR5-mCherry, mCherry was produced in the 4T1-IDO1 media but not in the 4T1-EV media (Fig. 3F).

### Kynurenine-responsive genetic circuits enabled tuning *S. enterica* growth in response to kynurenine at the physiological levels present in tumors

With the pKynR5 dual plasmid system in hand, we used it to control *S. enterica* growth in response to kynurenine through knocking out a gene needed for *S. enterica* growth and supplying a copy of this gene under pKynR5 control. We first chose the *asd* gene, which is critical for synthesizing diaminopimelic acid (DAP) needed for making the bacterial peptidoglycan layer (*51*). Because DAP is not made by mammalian cells, *S. enterica* is unable to survive in vivo without expressing the *asd* gene. Our initial attempts, however, were not very successful as *S. enterica* was able to grow in minimal media that lacked DAP even without kynurenine supplementation, suggesting a leak in *asd* gene expression in the OFF status. Consequently, we further modified our system through modifying the RBS of the *asd* gene and including a degradation tag (LAA) at its 3′ end (*52*). These modifications led to the *S. enterica* Δ*asd*/pkynR5-asd mutant, which showed controlled growth in response to kynurenine (fig. S6A). In parallel, we took similar approaches with the *murI* gene, which is critical for the biosynthesis of d-glutamate (*53*), another monomer that is needed for making the bacterial cell wall and cannot be supplied by the mammalian cells (fig. S6B). To enhance *S. enterica* dependence on kynurenine, we made a double *S. enterica* knockout in both *murI* and *asd* genes and supplied both genes in an operon on plasmid B. We named this *S. enterica* mutant strain AD11+ (fig. S6C).

**Fig. 4.**
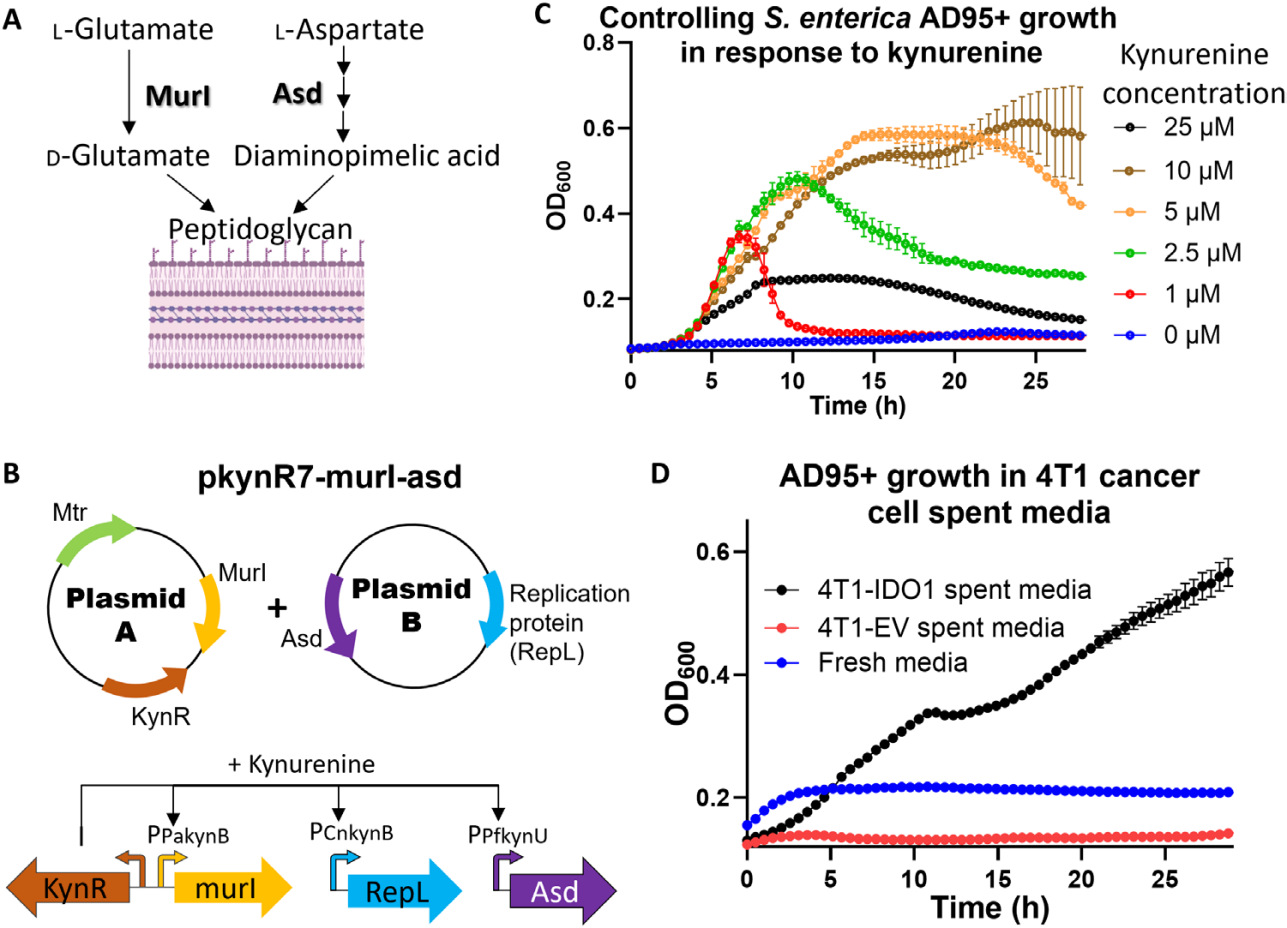
Engineering *S. enterica* to be dependent on kynurenine for growth. (**A**) Asd and MurI enzymes are essential for biosynthesis of d-glutamate and DAP, respectively, two key components for bacterial peptidoglycan cell wall formation. (**B**) Schematic diagram for the modified pKynR7 dual plasmid system used to generate the *S. enterica* AD95+ strain (*S. enterica* Δ*murI*Δ*asd*/pkynR7-murI-asd). (**C**) Growth kinetics of mutant AD95+ in M9 media at increasing kynurenine concentrations. M9 media were supplemented with 0.4% glucose and 0.1% casamino acids. h, hours. (**D**) Both 4T1-IDO1 and 4T1-EV were cultured in DMEM-10% serum media, and spent media were aliquoted in a 96-well plate. The kynurenine-controlled *S. enterica* AD95+ strain was added to different wells, and bacterial growth was monitored. Plotted are the means ± SE. *n* = 3 to 4. Created in BioRender. Dwidar, M. (2025) https://BioRender.com/d55t198.

To reduce the potential emergence of escaping kynurenine-independent mutants, we optimized the system to supply the *asd* gene on plasmid B while supplying *murI* on plasmid A of the dual plasmid system so that both genes are controlled by kynurenine-responsive promoters (Fig. 4, A and B). This improved kynurenine-dependent *S. enterica* Δ*murI*Δ*asd*/pkynR7-murI-asd is referred to hereafter as “AD95+.” Testing AD95+ in minimal media showed tight control of growth in response to kynurenine (Fig. 4C) with no growth at a 0 μM kynurenine concentration and maximum growth at 1 to 10 μM, the typical concentration observed in tumors (Fig. 1). The reduction in optical density at 600 nm (OD_600_) observed after the exponential growth phase is presumably due to the lysis of the bacterial cells as a result of consumption of a key nutrient. Previous studies found that bacteria like *E. coli* and *Mycobacterium tuberculosis* metabolize kynurenine through transaminases into kynurenic acid (*54*, *55*). When wild-type *S. enterica* was cultured in M9 media supplemented with kynurenine, kynurenine was metabolized into kynurenic acid (fig. S7). Kynurenine consumption may result in the inability to synthesize the cell wall monomers (i.e., DAP and d-glutamate) during the active growth phase, leading to cell lysis.

Last, kynurenine-dependent *S. enterica* AD95+ could grow only in the 4T1-IDO1 spent media (which contained kynurenine) but not in 4T1-EV spent media or fresh Dulbecco’s modified Eagle’s medium (DMEM)-10% serum media (Fig. 4D). Because each of the two plasmids in our system (which harbor kanamycin versus chloramphenicol resistance markers) contains one gene that is critically needed for survival (*asd* and *murI*), the engineered *S. enterica* cannot lose either of them. This was confirmed through culturing AD95+ in the presence of kynurenine without antibiotics followed by plating on plain versus antibiotic-supplemented plates. The results found a similar number of colonies, indicating the stability of both plasmids in AD95+ (fig. S8).

### *S. enterica* AD95+ shows superior tumor specificity in preclinical murine models for OC and TNBC when compared to VNP20009 strain

Several previous studies found that administering unattenuated wild-type *S. enterica* leads to severe toxicity and death of the mice within few days (*56*–*58*). Consistent with these studies, when wild-type *S. enterica* American Type Culture Collection (ATCC) 14028s were intraperitoneally injected into four subcutaneous tumor-bearing mice, one mouse died within 2 days before the predetermined endpoint, while the three remaining mice exhibited signs of severe systemic infection including hunching, grimace, and lethargy. Euthanizing these mice 2 days after bacterial injection, we observed a high number of bacteria in the liver and spleen as expected, with a tumor-to-liver ratio of only 159-fold (fig. S9). Consequently, we chose to use *S. enterica* VNP20009 as a control. The VNP20009 strain is a derivative of *S. enterica* ATCC 14028s, which was developed through chemical and ultraviolet mutagenesis, leading to its attenuation by purine auxotrophic mutation followed by knocking out of the *msbB* gene in a subsequent study to render its lipid A less immunogenic (*9*)*.* VNP20009 is one of the best characterized and successful *S. enterica* mutants in terms of specificity to tumors. Previous studies in mice found that VNP20009 accumulates in tumors at numbers up to 1000-fold higher than the numbers in the liver, spleen, and other organs (*10*, *59*). Our initial experiments testing VNP20009 via intraperitoneal injection in KPCA.A subcutaneous murine tumor models confirmed its preferential accumulation in tumors with the tumor-to-liver ratio close to 1000-fold (fig. S10).

**Fig. 5.**
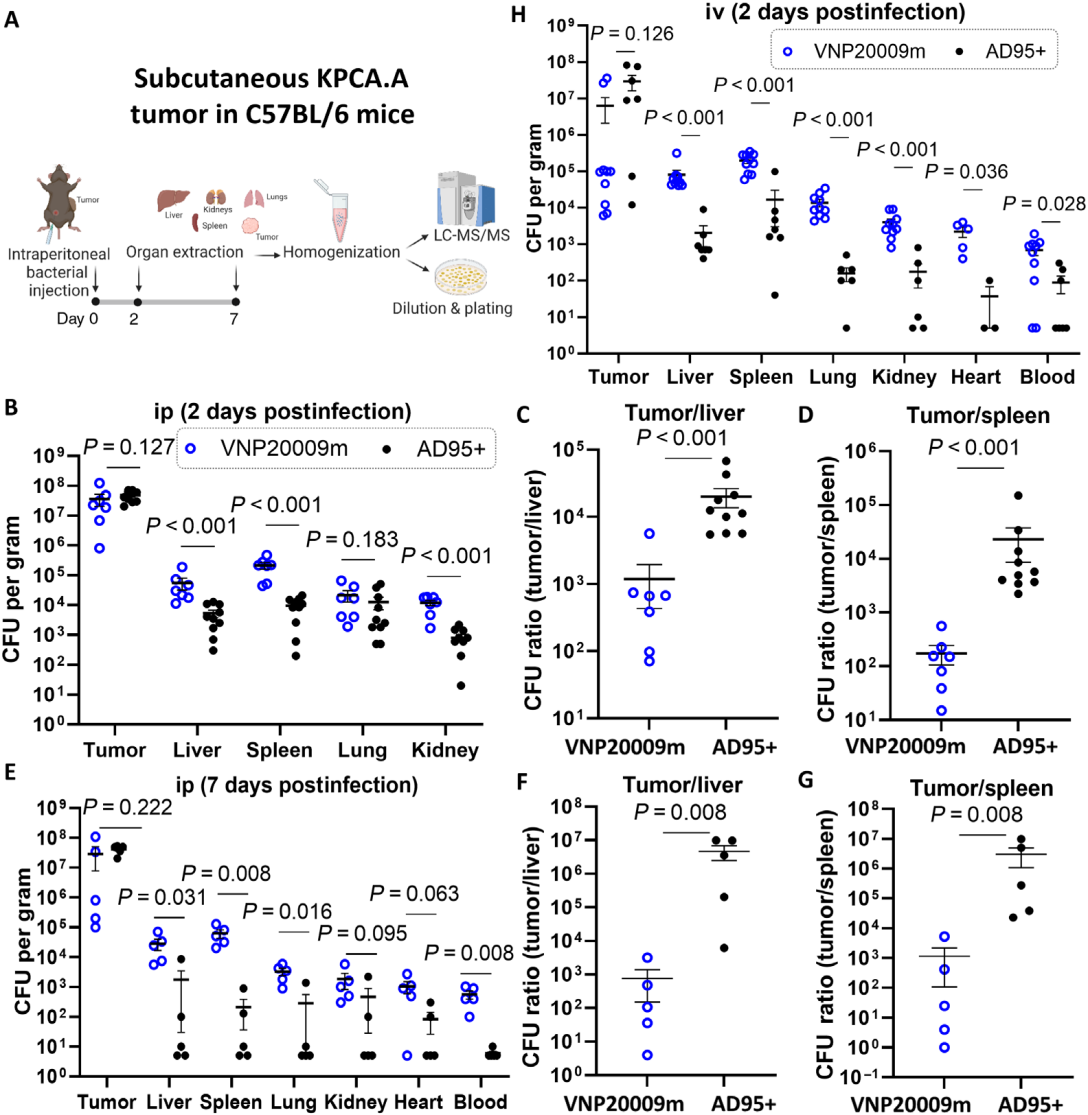
AD95+ has superior specificity to KPCA.A tumors compared to VNP20009m. (**A**) Experimental scheme. (**B** to **D**) Subcutaneous KPCA.A tumors were injected in C57BL/6 mice. Once tumors reached an average size of roughly 800 mm^3^, *S. enterica* mutants were intraperitoneally injected at a dose of 2 × 10^6^ to 4 × 10^6^ CFU. Mice were euthanized 2 days later, and organs were harvested for CFU counting. *n* = 7 to 10. Experiments were repeated on different days. Graphs depict cumulative data, with each symbol representing results from individual mouse. ip, intraperitoneally. (**E** to **G**) The same experiment was repeated; however, organs were analyzed 7 days after intraperitoneal bacterial injection. (**H**) Experiment was repeated via intravenous injection of bacteria. Lines represent the means ± SE. When no colonies were detected at the highest dilution, the number was stated as 5 CFU/g tissue for statistical analyses, which is half the limit of detection. iv, intravenously. The Mann-Whitney test was used in statistical analysis. *P* values are presented in panels. Created in BioRender. Dwidar, M. (2025) https://BioRender.com/d55t198.

To enable more accurate quantification of VNP20009 in the tissues, we transformed this strain with a chloramphenicol resistance plasmid to generate VNP20009m so that it can be plated and counted on agar plates supplemented with chloramphenicol. This was done to reduce the possible false counting of contaminating bacterial colonies. To ensure the stability of this chloramphenicol resistance plasmid in VNP20009m, it was supplied with a *murI* gene copy, which was knocked out from the VNP20009m chromosome. The AD95+ strain likewise contains the chloramphenicol resistance plasmid. We then compared our engineered AD95+ strain with VNP20009m in subcutaneous KPCA.A tumor models. When the tumors grew to a size of ~600 mm^3^ on average, AD95+ or VNP20009m was injected intraperitoneally at a dose of 2 × 10^6^ to 4 × 10^6^ colony-forming units (CFU). Mice were euthanized 2 days later, and tissues were harvested to determine bacterial distribution in tumors versus liver, spleen, and other organs through plating on LB plates (supplemented with d-glutamate and DAP) (Fig. 5A). The results indicate that both strains proliferate and accumulate in tumors to similar degrees (Fig. 5B). AD95+ exhibited higher tumor specificity compared to VNP20009m in terms of lower distribution to other organs with averages of roughly 19,000:1 and 23,000:1 for tumor/liver and tumor/spleen ratios, respectively (Fig. 5, C and D). Similar results were also obtained when organs were analyzed at 7 days postintraperitoneal bacterial injection (Fig. 5, E to G).

To further confirm the superior tumor targeting of AD95+, we opted next to test the distribution after intravenous injection. Compared to the intraperitoneal route, intravenous injection leads to faster distribution to distant tissues. The results showed that intravenous injection of AD95+ (via a retro-orbital route) leads again to higher tumor accumulation compared to VNP20009m, consistent with intraperitoneal injection at the 2-day time point (Fig. 5H). The higher tumor specificity for AD95+ was even more evident at 7 days after intravenous injection (fig. S11, A to C) with nearly complete absence of AD95+ in all organs, except tumors at this time point. This was accompanied by smaller spleen weights when compared to mice injected with VNP20009m (fig. S11D). The better tolerance for AD95+ compared to VNP20009m was also evident from the reduced levels of several plasma cytokines including IFN-γ, interleukin-1β (IL-1β), IL-2, KC/GRO, IL-10, and TNFα (fig. S11, E to N). Many of these cytokines are known to be elevated in sepsis (*60*) and are associated with cytokine release syndrome (*61*). Last, direct intratumor injection of both strains confirmed that AD95+ accumulates in KPCA.A tumors with limited diffusion to other organs in comparison to the VNP20009m strain (fig. S12).

**Fig. 6.**
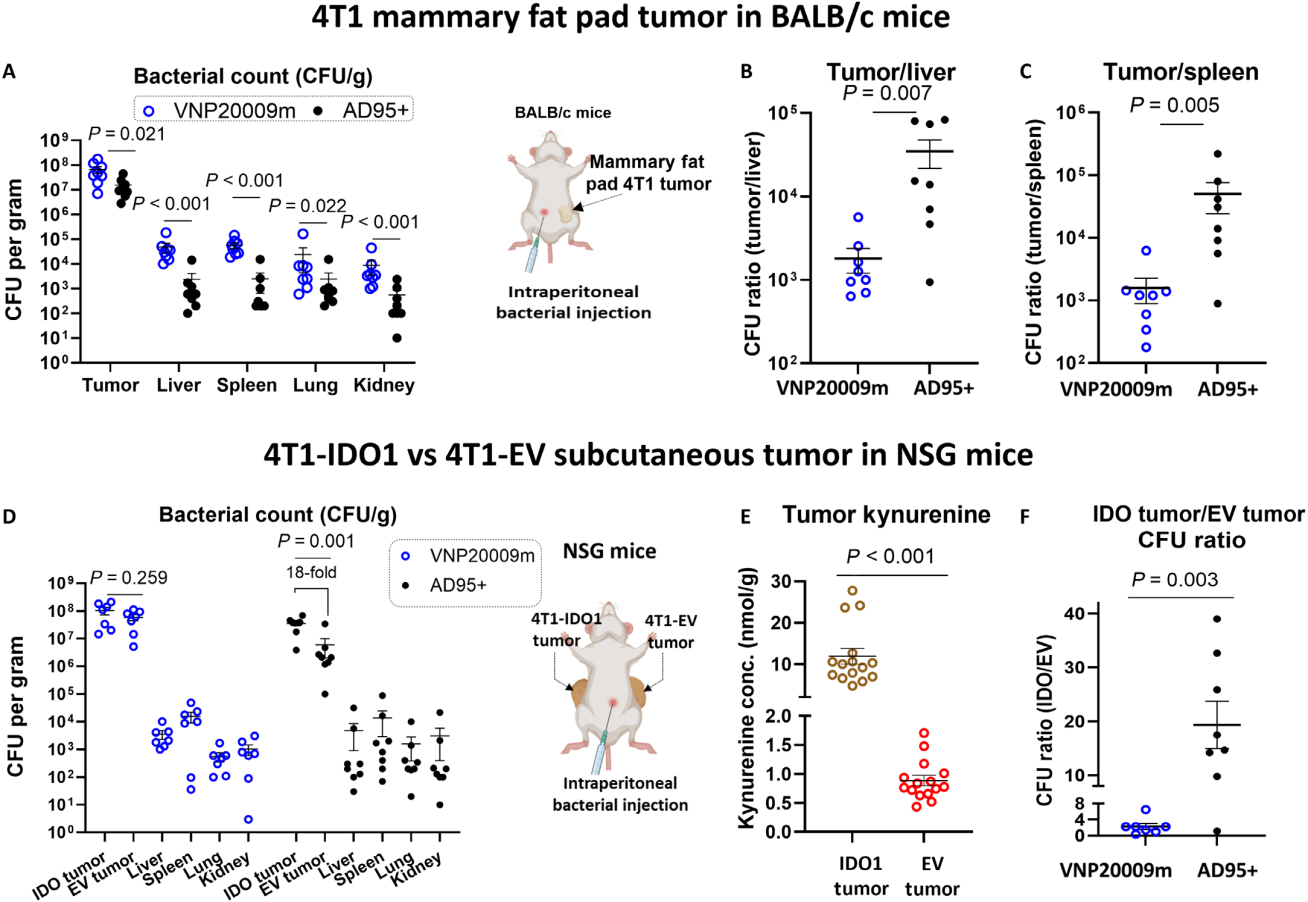
AD95+ cancer specificity depends on tumor kynurenine content. (**A**) 4T1 tumors were established in the mammary fat pads of BALB/c mice. When tumors reached an average size of ~500 mm^3^, *S. enterica* mutants were intraperitoneally injected at a dose of 2 × 10^6^ to 4 × 10^6^ CFU. Mice were euthanized 2 days later, and organs were harvested for CFU counting. (**B** and **C**) CFU count in tumor versus spleen and liver for each strain showing that engineered AD95+ has significantly higher tumor specificity compared to VNP20009m. (**D** to **F**) 4T1-IDO1 and 4T1-EV tumors were injected on opposite flanks in NSG mice. When tumors were roughly 300 to 400 mm^3^, *S. enterica* AD95+ or VNP20009 was injected intraperitoneally (2 × 10^6^ CFU). Mice were euthanized 2 days later, and kynurenine and number of bacteria in each tumor were determined through LC-MS/MS and plating, respectively. *n* = 7 to 8 per group. Lines represent the means ± SE. For statistical analyses, when no colonies were detected at the highest dilution, the number was stated as 5 CFU/g tissue, which is half the limit of detection. Statistical analysis was done by the Mann-Whitney test. *P* values are presented in panels. Created in BioRender. Dwidar, M. (2025) https://BioRender.com/d55t198.

The two strains were then compared through intraperitoneal injection in the orthotopic 4T1 TNBC model injected in the mammary fat pads of BALB/c mice. AD95+ demonstrated high tumor specificity (Fig. 6, A to C). AD95+ showed high selectivity for tumors including those with low kynurenine concentrations (i.e., less than 1 nmol/g; fig. S13, A and B).

To confirm that AD95+ tumor preference depends on kynurenine, immunocompromised NSG mice were injected with 4T1-IDO1 and 4T1-EV cells on opposite flanks within the same mice. NSG mice have partial impairment in innate immunity and complete ablation of adaptive immunity (*62*). Using NSG mice in this experiment, therefore, minimizes immune-induced kynurenine accumulation in the control 4T1-EV tumors. Both cell lines developed tumors at comparable rates. AD95+ and VNP20009m were injected intraperitoneally to determine relative homing to both tumors. The results indicate that AD95+ homing to tumors is dependent on kynurenine with an average of 18-fold difference in accumulation in 4T1-IDO1 versus 4T1-EV tumors (Fig. 6, D to F). As expected, VNP20009 showed no significant difference in distribution between 4T1-IDO1 and 4T1-EV tumors (Fig. 6, D to F). Our engineered AD95+ strain still retained relatively high specificity to the 4T1-EV tumors (~1000-fold) when compared to its distribution in other organs (liver, spleen, kidneys, and lungs) (Fig. 6D), indicating that the specificity to the tumor is not purely dependent on kynurenine but in part due to also the natural tendency of *S. enterica* to accumulate in tumors. This also underscores the potential of this engineered strain to target tumors even if their kynurenine concentration is as low as 0.5 nmol/g (Fig. 6E and fig. S13C).

### Kynurenine-dependent *S. enterica* AD95+ attenuates tumor growth

**Fig. 7.**
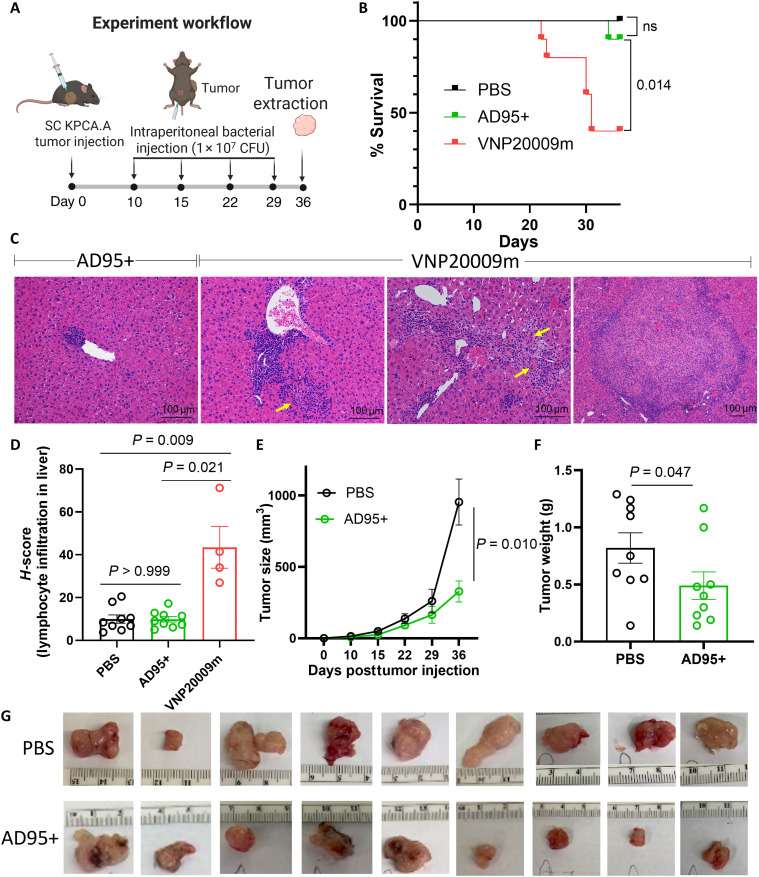
At high doses, AD95+ is better tolerated than VNP20009m and reduces tumor growth. (**A**) Experimental design. KPCA.A tumors were injected subcutaneously in C57BL/6 mice. Once tumors were detected, *S. enterica* mutants AD95+ and VNP2009m were intraperitoneally injected at a dose of ~1 × 10^7^ CFU weekly. Tumor growth and mice survival were monitored over time. (**B**) During the experiment, 6 of 10 mice in the VNP20009m group while only 1 of 10 AD95+-treated mice died. All remaining mice were euthanized 36 days after the tumor injection. *n* = 9 to 10 per group. ns, not significant. (**C**) Livers were harvested from mice at the endpoint, formalin fixed, and H&E stained. Histology analyses revealed significant leukocyte infiltrates (predominantly lymphocytes) in livers from mice treated with VNP20009m, while PBS- and AD95+-treated mice showed only mild or no infiltration. The leftmost image shows a representative minor infiltration by mononuclear leukocytes (>100 μm) surrounding one injured hepatocyte in the liver from mouse treated with AD95+. Infiltrates in mice treated with VNP20009m exceed 100 μm and span liver lobules (middle images). Yellow arrows indicate nodules of necrotic hepatocytes. The rightmost image shows representative large areas of necrosis surrounded by mixed leukocyte infiltrates in mice treated with VNP20009m. (**D**) Liver infiltration by lymphocytes for each group was quantified as described in Materials and Methods. Heatmaps representing lymphocyte infiltration are shown in fig. S14. (**E**) Tumor growth was monitored weekly via palpation. (**F**) Tumors were harvested at the endpoint, and mass was obtained. AD95+-treated mice exhibit significantly smaller tumors. Plotted are the means ± SE. (**G**) Images of the excised tumors at the end of the study. Tumors from surviving mice were all harvested on the same day (day 36 after tumor injection). Created in BioRender. Dwidar, M. (2025) https://BioRender.com/d55t198.

To investigate whether the *S. enterica* AD95+ strain can reduce tumor growth, we established subcutaneous KPCA.A tumors in C57BL/6 mice. Once tumors were palpable, we administered weekly intraperitoneal injections of either vehicle [phosphate-buffered saline (PBS)] or bacteria at a dose of 1 × 10^7^ CFU (Fig. 7A). Tumor growth was monitored by palpation over a 5-week period. Nine of 10 AD95+-treated mice survived and tolerated the treatment until the endpoint (Fig. 7B) equivalent to PBS, albeit with smaller tumors. VNP20009m was evaluated as a benchmark. However, we determined that weekly intraperitoneal treatment with VNP20009m at this dose (1 × 10^7^ CFU) was toxic with only four mice surviving to the endpoint (Fig. 7B). We analyzed liver, spleen, and kidney specimens from mice treated with PBS, AD95+, or VNP20009m. No significant changes were present in the kidneys. Spleens demonstrated no changes in the white pulp and expansion of red pulp with extra medullary hematopoiesis in both groups treated with bacteria. The histology of the livers from mice treated with AD95+ was not significantly different from the control PBS group. In contrast, there was significant infiltration by mixed leucocytes in the livers from the VNP20009m group. The livers had widespread aggregates of predominantly lymphocytes that frequently exceeded 100 μm and spanned liver lobules. These infiltrates surrounded nodules of necrotic hepatocytes (Fig. 7, C and D, and fig. S14). We also determined that AD95+ suppresses tumor growth (Fig. 7E) and tumor mass compared to PBS-treated mice (Fig. 7, F and G). These studies are consistent with the concept that AD95+ can specifically home to the tumor and lead to tumor growth inhibition.

**Fig. 8.**
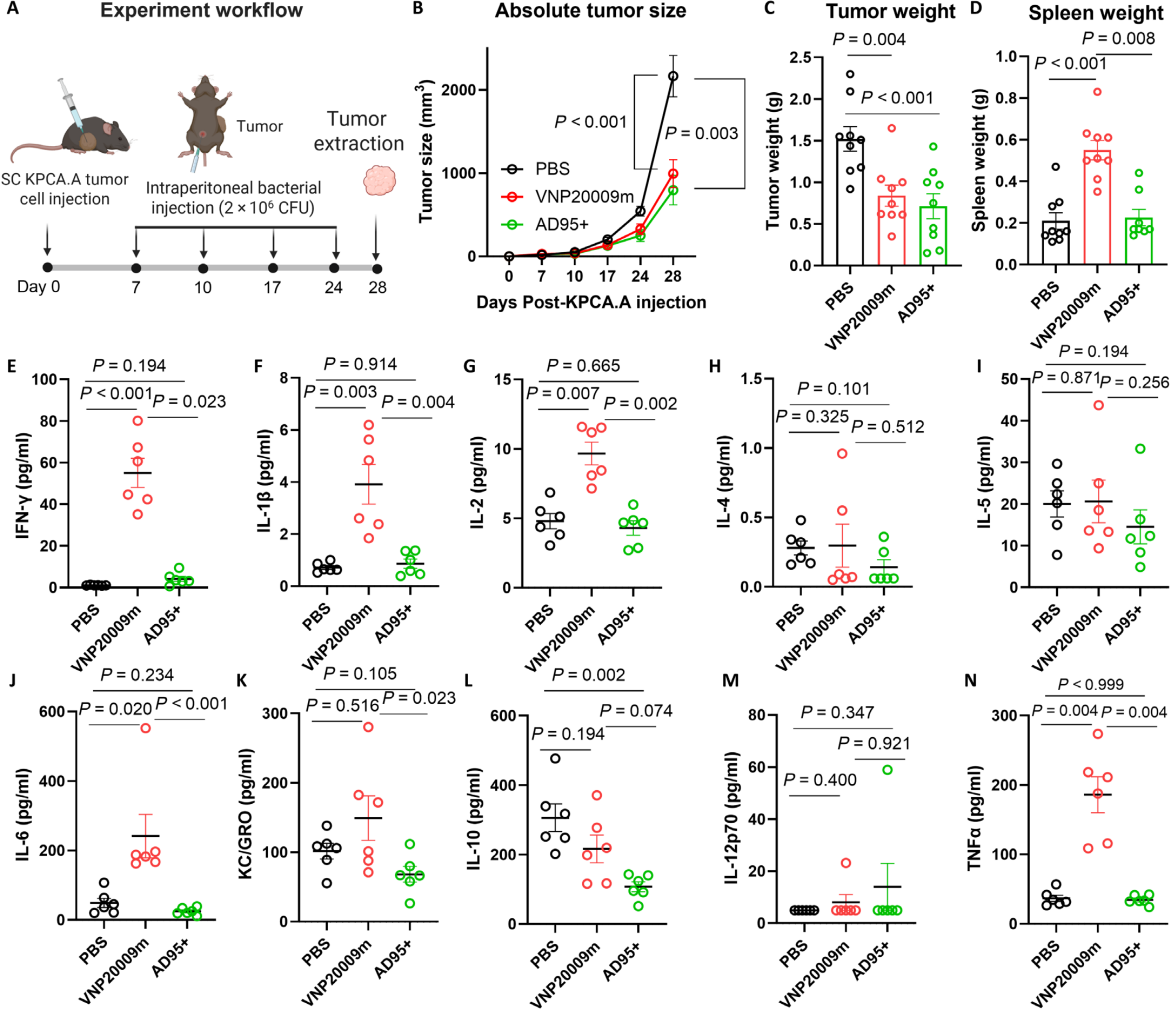
At low doses, AD95+ attenuates tumor growth and results in less systemic toxicity when compared to VNP20009m. (**A**) Experimental design. KPCA.A tumors were injected subcutaneously in C57BL/6 mice. Once tumors were detected, *S. enterica* mutants AD95+ and VNP2009m were intraperitoneally injected at a dose of ~2 × 10^6^ CFU weekly. Tumor growth and mice survival were monitored over time. All mice were euthanized 28 days after the tumor injection. *n* = 9 per group. Plotted are the means ± SE. (**B**) Tumor growth was monitored weekly via palpation. (**C**) Tumors were harvested at the endpoint, and mass was obtained. (**D**) Spleen weights at the endpoint. (**E** to **N**) Blood was harvested from both groups at the endpoint, processed for plasma, and analyzed for levels of inflammatory cytokines. IL-4 and IL-12p70 were detected in some but not all samples in our assay. When not detected, the values were stated at 0.06 and 4.98 pg/ml, which are corresponding to half the lower limit of detection for each, respectively. Plotted are the means ± SE. Statistical significance was tested using Kruskal-Wallis test. Created in BioRender. Dwidar, M. (2025) https://BioRender.com/d55t198.

To further confirm the therapeutic efficacy of AD95+, we repeated the experiment with a lower dose (2 × 10^6^ CFU) for AD95+ and VNP20009m (Fig. 8A). The results showed that mice injected with either of the two strains had significantly slower tumor growth and smaller tumors at the endpoint compared to control PBS-injected mice (Fig. 8, B and C). We did not observe mortalities because of bacterial infection at this dose in any of the groups. Nevertheless, mice injected with VNP20009m showed a significantly higher degree of splenomegaly at the endpoint compared to the PBS- or AD95+-injected mice (Fig. 8D). These results are consistent with the concept that treatment with AD95+ exhibits lower toxicity to the spleens compared to VNP20009m. The lower toxicity for the AD95+ group is also consistent with the similar plasma cytokine profile as the PBS group (Fig. 8, E to N). Except for the anti-inflammatory IL-10, which was higher in the PBS group, no significant differences were found between PBS and AD95+ groups for any of the tested cytokines. In contrast, VNP20009m-treated mice showed significantly elevated levels of different cytokines including IFN-γ, IL-1β, IL-2, IL-6, KC/GRO, and TNFα (Fig. 8, E to N). We also noticed that VNP20009m, but not AD95+, repeated administration over prolonged time (Fig. 8) resulted in some lethargy and general distress to the mice. The difference between the two strains was more evident when administered at high doses (1 × 10^7^ CFU) in Fig. 7.

## DISCUSSION

Relying on the natural tendency of some bacteria like *S. enterica* to preferentially accumulate in solid tumors is the core principle of bacterial-based cancer therapy. Despite this natural tropism for tumors, there remains a need to improve tumor bacterial specificity. Previous efforts to engineer tumor-specific bacteria resulted in *S. enterica* VNP20009, which showed high specificity in murine models (~1000-fold tumor-to-liver ratio) (*10*). VNP20009 was used as a benchmark in our studies. Even with this promising level of tumor site accumulation, this strain was not successful in phase 1 clinical trials (*11*). Other groups aimed to target tumor hypoxia through using obligate anaerobic bacteria such as clostridia, which germinate only in anaerobic microenvironments (*63*). These approaches, however, limit the bacterial growth to the tumor core while leaving the outer rim of the tumor unaffected (*63*, *64*). Here, we sought an alternate unique approach to enhance the specificity of *S. enterica* to tumors by targeting tumor-enriched kynurenine. A previous study (*24*) showed that most solid tumors exhibit variable levels of IDO1 overexpression. The proportion of tumor cells with IDO1 overexpression, however, is highly variable between different tumors. In that study, 8 of 10 ovarian tumor specimens were positive for IDO1 overexpression. In our current study, we found that kynurenine production is variable between samples of the same tumor type. In TNBC, three of three specimens exhibited kynurenine levels more than fourfold higher than the healthy tissue levels. In OC, 11 of 15 specimens showed high kynurenine levels with 8 of 15 specimens showing kynurenine levels more than fivefold higher than the average benign ovarian tumor level (Fig. 1). With this background in mind, we constructed bacterial kynurenine-responsive genetic circuits that allow control of gene expression and growth in bacteria (including *E. coli* and *S. enterica*) in response to kynurenine secreted by solid tumors. Using this circuit, we demonstrated high bacterial specificity to tumors, largely driven by kynurenine. The high specificity of the bacteria to tumors appears to lead to better tolerance by the mice and less mortality when compared to the benchmark strain VNP20009, as evident from better survival, less splenomegaly, and lower plasma cytokines (Figs. 7 and 8 and fig. S11).

Although we focused here on two cancer types (OC and TNBC), our approach is applicable to various solid tumors as kynurenine accumulation is reported in almost all types of solid tumors (*20*–*26*). One limitation of our study is that kynurenine is not produced equally by all solid tumors and depends on the degree of T cell and IFN-γ infiltration within the tumor microenvironment (*30*). Nevertheless, the AD95+ mutant could grow with high specificity in tumors with only low kynurenine concentration (0.5 nmol/g) (Figs. 5 and 6 and figs. S12 and S13). Given the heterogeneous nature of solid tumors and the uneven spatial distribution, some studies suggest that the actual kynurenine concentration in some locations within the tumor microenvironment is higher than the average kynurenine concentration in the bulk tumor tissue (*65*). This could explain how AD95+ accumulates in tumors with low bulk kynurenine concentration.

In conclusion, through using kynurenine as a cancer-specific cue, the present study lays the foundations for enhanced specific bacterial targeting to the tumors rather than healthy tissues—a critical needed step for clinical application of bacterial-based cancer therapy. Our engineered kynurenine-controlled AD95+ strain may prove useful in future work as a next-generation drug delivery chasis for a variety of therapeutic anticancer payloads including enzymes to locally activate chemotherapeutic prodrugs into active drugs (*66*–*68*), immunomodulatory agents (*69*), and anticancer nanobodies (*70*, *71*) and has potential as a superior alternative to the current tumor-targeting *S. enterica* strains. Furthermore, applications of engineered kynurenine-controlled genetic circuits as both therapeutics and diagnostics may extend beyond cancer to other inflammatory diseases in which kynurenine is also overproduced including inflammatory bowel diseases (*72*). In addition, more precise tumor targeting can be achieved in the future through combining kynurenine-based control with other targeting strategies such as purine auxotrophy or hypoxia- and acidity-driven bacteria targeting (*52*, *73*, *74*).

## MATERIALS AND METHODS

### Human breast and ovarian tumor specimen collection

Human ovarian tumor and benign specimens were collected from consented patients undergoing surgery at the Cleveland Clinic Foundation using Institutional Review Board (IRB) protocol no. 19-185. Discarded healthy breast and tumor tissues were obtained from surgical pathology immediately after surgery. The tissues used for this study are not considered essential for diagnosis or treatment decisions and would otherwise be discarded. Tumors were snap frozen and stored for processing.

### Microbial strains and culturing conditions

*E. coli* Steller (Clontech), *S. enterica* ATCC 14028s, and *S. enterica* VNP20009 (ATCC BAA-3199) were used for all experiments. All were routinely streaked on LB (Lauria Bertani; Difco) agar plates and cultured in LB broth. For *S. enterica* mutants lacking *asd* and *murI* genes, DAP and d-glutamate were added to the cultures at 250 μg/ml for each. When needed, ampicillin, kanamycin, and chloramphenicol were added at 100, 50, and 17 μg/ml, respectively.

### Cloning *kynR* and its cognate promoters and optimizing the performance in *E. coli*

For initial experiments, the DNA sequence spanning the *kynR* gene together with its nearby cognate promoter upstream of the *kynB* gene was amplified from *P. aeruginosa* MRSN1583 through polymerase chain reaction (PCR) and placed upstream of the *mCherry* reporter on plasmid pPaKynR1-mCherry. The RBS sequences were optimized for *kynR* and *mCherry* in pPaKynR2-mCherry using the RBS calculator tool (https://salislab.net/software/predict_rbs_calculator) (*75*, *76*). To clone the aromatic amino acid transporters, Mtr, AroP, and TnaB, each was PCR amplified from the *E. coli* MG1655 genome and placed downstream of the PJ23115 promoter (https://parts.igem.org/Part:BBa_J23115) to make pMtr-PakynR2-mCherry, pAro-PakynR2-mCherry, and pTna-PakynR2-mCherry plasmids, respectively. The PJ23115 promoter was then changed for the PJ23113 promoter in the p113-Mtr-PakynR2-mCherry plasmid. To compare kynurenine-responsive promoters from various bacteria, the promoter sequence upstream of the *kynU* or *kynB* gene in each strain was PCR amplified from the corresponding genome and placed upstream of *mCherry* in a series of plasmids (pKynR-Pa-mCherry, pKynR-Pf-mCherry, pKynR-Cn-mCherry, pKynR-Bt-mCherry, pKynR-Bc-mCherry, pKynR-Bp-mCherry, pKynR-Rs-mCherry, and pCerKynR1-mCherry). These plasmids contained the *kynR* gene from *P. aeruginosa* downstream of the PJ23114 promoter. In these plasmids, the *gfp* gene was included upstream of each of the tested promoters in an opposite direction. This was done to test for induction of expression in the opposite direction upon inducing each of the tested promoter sequences with kynurenine. None of the tested promoters induced green fluorescent protein expression, indicating that all tested sequences are unidirectional promoters. Figure S2 illustrates the sequence of each of the cloned sequences and the consensus kynurenine-responsive promoter sequence.

To construct the optimized dual pkynR5-mCherry system, *kynR* and *mtr* transporter genes were kept on plasmid A under the control of the PJ23113 and PJ23114 promoters, respectively, (plasmid A, p113m-114kynR). The reporter *mCherry* (or *nluc* gene) was placed in another plasmid (plasmid B, pTrig-cn-repL-pf-cherry). The plasmid B backbone was modified from our previous study (*46*) through placing the *C. necator* P_kynB_ promoter sequence upstream of the *repL* gene, while the *P. fluorescens* P_kynU_ promoter sequence was placed upstream of the *mCherry* gene. All constructed plasmids were transformed into *E. coli* Steller through chemical transformation and verified through Sanger sequencing. Transformation of *S. enterica* was done through electroporation using the purified plasmid. Figure S15 illustrates the maps of key constructed plasmids. Tables S1 and S2 show all the plasmids and bacterial strains constructed during the course of the study, respectively.

### Assessing growth, mCherry fluorescence, and nLuc bioluminescence of *E. coli* and *S. enterica*

*E. coli* and *S. enterica* strains harboring the respective plasmids were cultured in LB broth for 24 hours. Two microliters of these suspensions were added to 200 μl of M9 media supplemented with glucose at 0.4% inside 96-well plates. LB and M9 media were supplemented with chloramphenicol and kanamycin when appropriate. The plates were incubated at 37°C with shaking within a Tecan Infinite microplate reader. OD_600_ and mCherry fluorescence (580 nm/610 nm) were recorded at 30-min intervals over 24- to 48-hour periods. mCherry fluorescence for each strain was calculated as (mCherry fluorescence of test strain/OD_600_) − (Background fluorescence of control strain/OD_600_). nLuc bioluminescence was measured after 24 hours of incubation using the Nano-Glo Luciferase Assay kit (Promega) according to the manufacturer’s instructions.

For experiments assessing the kynurenine-dependent growth of mutant *S. enterica* strains, M9 media were supplemented with both glucose at 0.4% and casamino acids at 1%. For these experiments, it was necessary to incubate the LB starter cultures of *S. enterica* AD95+ for 24 hours followed by centrifugation and washing of the pellets twice in M9 media. We noticed that traces of LB media allowed the growth of *S. enterica* AD95+ mutant in the absence of externally added kynurenine. The suspensions were then diluted 10-fold in M9 media, and then 2 μl of these suspensions were added to 200 μl of M9 supplemented with glucose at 0.4% and casamino acids at 1% inside 96-well plates.

### Knocking out *asd* and *murI* genes in *S. enterica*

For gene knockout in *S. enterica* ATCC 14028s, bacteria were first rendered ampicillin resistant through transformation with plasmid p101-Amp containing an ampicillin-resistance cassette and a temperature-sensitive derivative of the pSC101 replication origin. This plasmid was constructed through deleting λ red recombination genes from the pKD46 plasmid (*77*). Two PCR reactions were then used to amplify ~1-kb DNA fragments surrounding the sequence to be deleted in the *S. enterica* chromosome. The two homologous recombination arms were fused through a third PCR reaction and then ligated to a suicide plasmid using the In-Fusion HD Cloning kit (Clontech). The suicide plasmid contained R6K replication origin, RP4-oriT, *sacB* as a counter selection marker, and a kanamycin-resistance cassette. The ligated suicide plasmid was transformed into *E. coli* S17 λpir and then transferred to *S. enterica* through conjugation. The resulted *S. enterica* merodiploid conjugants were plated on LB agar plates containing kanamycin at 50 ng/μl and carbenicillin at 200 ng/μl. One *S. enterica* merodiploid mutant was then selected and restreaked on an LSW-Sucrose agar plate (*78*). The composition of the LSW agar media was tryptone (10 g/liter), yeast extract (5 g/liter), glycerol (5 ml/liter), NaCl (0.4 g/liter), sucrose (100 g/liter), and agar (20 g/liter). The plates were supplemented with kanamycin at 50 ng/μl and either d-glutamate at 250 μg/ml (for *murI* knockouts) or DAP at 250 μg/ml (for *asd* knockouts). Screening for knockout mutants was done through PCR. The correct knockouts were selected, restreaked, and confirmed for the loss of the conjugated plasmid through DNA sequencing, its inability to grow in the presence of kanamycin, and its auxotrophy for the respective metabolite (d-glutamate or DAP). To cure the knockouts from the ampicillin-resistance plasmid, they were cultured at 40°C and screened for the loss of ampicillin resistance.

To knock out *murI* in *S. enterica* VNP20009, we used the same process, except that we had to transform it with the p101-Amp-murI plasmid containing an extra copy of the *murI* gene before knocking out the chromosomal *murI* gene. After knocking out the chromosomal *murI* gene, the VNP20009 Δ*murI* mutant was cured from plasmid p101-Amp-murI through culturing on LB plates supplemented with d-glutamate in the absence of ampicillin at 40°C. To construct the *S. enterica* VNP20009m strain, the *murI* gene was supplemented in trans under the constitutive promoter PJ23113 on a plasmid (pMurI) harboring also a chloramphenicol-resistance cassette and pBR322 origin.

### Mass spectrometry analyses for kynurenine in mouse tissues and cell cultures

Mouse tissues were minced with a scalpel. Fifty- to 200-mg portions of the minced tissues were mixed with 3 volumes of distilled water in 2-ml Eppendorf tubes. The suspensions were heated at 95°C to deactivate the enzymes in the kynurenine pathway and then homogenized using a bead tissue homogenizer (MM400, Retsch). All samples were then filtered through a 3-kDa cutoff membrane filter (Amicon, UFC5003BK) to remove the proteins and high-molecular-weight components. Aliquots (25 μl) were mixed with 2.5 μl of internal standard solution containing 100 μM of each of [^2^H_4_]-l-kynurenine (cat. no. D-8026, CDN isotopes) and [^2^H_5_]-l-tryptophan (cat. no. D-1522, CDN isotopes). Samples were then injected onto an LC-MS/MS spectrometer for quantitation. Two microliters of the prepared samples was injected onto an LC column through a Shimadzu autosampler (SIL-HTc). The metabolites were resolved on a C18 column (Prodigy, 150 by 2 mm, 5 μm, 00F-3300-B0, Phenomenex) with LC gradients generated from binary pumps (Shimadzu LC-20AD) connected to two solvents: A, 0.2% formic acid in water; B, 0.2% formic acid in methanol. The LC elutes were analyzed on an API 5000 Mass spectrometer (Sciex) with an electrospray ion source. Standards and internal standards were monitored in positive multiple reaction monitoring mode with parent-to-daughter ion transitions: mass/charge ratio (*m*/*z*) 209 → 94 for kynurenine, *m*/*z* 205 → 188 for tryptophan, *m*/*z* 213 → 96 for [^2^H_4_]-l-kynurenine, and *m*/*z* 210 → 192 for [^2^H_5_]-tryptophan. Mass spectrometry parameters were optimized for individual standards. Standard curves were generated from serial dilutions of standards undergoing the same procedures as real samples. For some of the earlier experiments, [^13^C_10_]-l-kynurenine (cat. no. CLM-9884, Cambridge Isotope Laboratories) was used as an internal standard instead of [^2^H_4_]-l-kynurenine. The parent-to-daughter ion transition for monitoring [^13^C_10_]-l-kynurenine was *m*/*z* 219 → 100. For experiments assessing the concentration of kynurenic acid, [^2^H_5_]-kynurenic acid (cat. no. D-439, CDN isotopes) was used as an internal standard. The parent-to-daughter ion transitions for monitoring kynurenic acid and [^2^H_5_]-kynurenic acid were *m*/*z* 189 → 144 and *m*/*z* 194 → 166, respectively.

### Cell lines and cell culture

All cell lines used in this study are summarized in table S3. Mouse ovarian epithelial cancer cell lines ID8 (*50*), KPCA.A (*39*), and BPPNM (*39*) (all are syngeneic with the C57Bl/6 mouse strain) were cultured in DMEM containing 5% heat-inactivated fetal bovine serum (FBS; Atlas Biologicals cat. no. F-0500-D), 1% insulin-transferrin-selenium (Thermo Fisher Scientific; ITS-G, 41400045), and 100 μl of epidermal growth factor (10 μg/ml) and grown under standard conditions (incubation at 37°C and 5% CO_2_). KPCA.A and BBPNM cell lines were a gift from R. A. Weinberg at Whitehead Institute for Biomedical Research to the Reizes lab (*39*). The mouse TNBC cell line 4T1-Luc (luciferase-expressing 4T1 cells, BALB/c syngeneic) was a gift from W. Schiemann at Case Western Reserve University. 4T1-Luc cells were routinely cultured in either RPMI media or DMEM containing 10% heat-inactivated FBS.

### Construction of 4T1-EV and 4T1-IDO1 breast cancer cell lines

The lentiviral vector harboring the murine *ido1* gene together with a fusion Luciferase-tdTomato gene (IDO1 vector) was constructed by VectorBuilder. A control empty vector that lacks the *ido1* gene (EV) was also constructed. Both vectors were purified from *E. coli* using the Plasmid Maxiprep kit (Nucleospin). Viral particle preparations were carried out in human embryonic kidney (HEK) 293T cells following a previously described method (*79*). Briefly, HEK293T cells were cultured in a 100-mm dish. When reaching 60% confluency, HEK293T cells were transfected with pMD2.G (lentiviral envelope-expressing plasmid), pRSV-Rev (lentiviral packaging plasmid), pMDLg/Prre (packaging plasmid containing Gag and Pol), and either the empty vector (EV) or the *Ido*1-expressing vector (IDO1) using Lipofectamine 3000 reagents (Thermo Fisher Scientific). After 24 hours, the transfection medium was removed, and fresh serum-enriched DMEM was added to the HEK293T cells. Following an additional 24 hours, DMEM containing lentiviral particles was filtered. The filtered media were added to 4T1-Luc cells. After 3 days, the media were changed, and regular DMEM-10% FBS media were added to the 4T1-Luc cells. Transduced cells were cultured, and flow cytometry was performed to isolate tdTomato-positive 4T1-Luc cells (top 10%). The sorted cells were cultured, stored as aliquots, and used for further experiments.

### Testing the *S. enterica* response to kynurenine in cancer cell culture supernatants

Cancer cell lines were cultured in 1 ml of DMEM within six-well plates at ~100,000 cells per well. IFN-γ (Peprotech, cat. no. 315-05) was added to the test wells at a concentration of 200 ng/ml. The plates were incubated for 72 hours under standard incubation conditions (37°C and 5% CO_2_). Culture supernatants were then collected, centrifuged, and processed for LC-MS/MS analyses. One hundred microliters of samples from the supernatants were then aliquoted in 96-well plates and used as culturing media for the engineered *S. enterica*. Overnight cultures of the engineered *S. enterica* harboring the pKynR5-mCherry dual plasmid system (or the control pKynR5-nLuc) were prepared in LB media and washed. Two microliters of the bacterial suspensions were then spotted in the wells containing the spent cancer cell supernatants. The plates were incubated within a Tecan Infinite microplate reader with shaking at 37°C. OD_600_ and mCherry fluorescence were measured. Data shown in Fig. 3 are at a 9-hour time point.

### Testing *S. enterica* cancer specificity in murine 4T1 and KPCA.A tumor models

All experiments involving mice were performed using Institutional Animal Care and Use Committee (IACUC) protocols approved by the Cleveland Clinic Animal Care and Use Committee (IACUC No. 2706, 3279, and 2924). For KPCA.A tumor models, cells were injected subcutaneously in the flank region in C57BL/6J mice. For 4T1 tumor models, cells were injected either in the flank or in the fourth mammary fat pads in BALB/c mice. For experiments using 4T1-IDO1 and 4T1-EV cell lines, they were injected subcutaneously on opposite flanks in immunocompromised NSG mice (*62*). *S. enterica* strains were grown overnight in LB media supplemented with DAP, d-glutamate, and chloramphenicol. Kanamycin was also added to the AD95+ cultures. At the day of the bacterial injection, bacterial cultures were washed and resuspended in PBS at an OD_600_ of 0.1. One hundred microliters of these suspensions were injected in mice either intraperitoneally, intravenously (via the retro-orbital route), or through direct intratumoral injection. Unless mentioned otherwise, the bacterial dose was equivalent to around 2 × 10^6^ to 4 × 10^6^ CFU based on colony counting on agar plates. Mice were anesthetized through isoflurane inhalation anesthesia during the injection and were monitored daily after the bacterial injection. For retro-orbital injection, a drop of ophthalmic anesthetic (0.5% proparacaine hydrochloride ophthalmic solution) was placed on the eye before injection. This provided additional procedural and postprocedural analgesia. At the specified days after bacterial injection, mice were euthanized through carbon dioxide asphyxia. Organs were extracted immediately after euthanasia. Each organ was cut into small pieces using a scalpel and then divided into two portions. One portion was snap frozen for later processing for LC-MS/MS analyses. The other portion was weighed, suspended in an equivalent volume of PBS, and homogenized using a bead homogenizer. The homogenate was diluted and plated on LB agar plates supplemented with d-glutamate, DAP, and chloramphenicol for colony counting. For statistical analyses, mice that showed <5 CFU/mg in the tumor samples were considered outliers and were excluded.

### Histology analyses

Sections from livers, spleens, and kidneys harvested from mice after euthanasia were immediately placed in tissue embedding cassettes and dipped in 10% buffered formalin overnight. The cassettes were then transferred to 70% ethanol solution, embedded in paraffin, sectioned, hematoxylin and eosin (H&E) stained, and examined by a pathologist. The slides were then imaged using an Aperio AT2 slide scanner (Leica Biosystems) at a resolution of 0.51 μm/pixel. Images of H&E-stained liver sections were analyzed using WSInfer extension (*80*) in QuPath (*81*) and a deep learning–based pretrained model (*82*) to detect the degree and distribution of lymphocyte infiltration. Images of the entire sections were divided into 25-μm by 25-μm squares. Each square was assigned a score between 0 and 1 for the lymphocyte infiltration followed by classification into four categories: high (H), medium (M), low (L), and negative (N). The predictions were reviewed by a pathologist followed by calculation of the *H*-score for each slide using the formula *H*-score = 100 × [(3 × #H) + (2 × #M) + (1 × #L)]/(#H + #M + #L + #N). Heatmaps representing the degree and distribution of the lymphocyte infiltration were generated in QuPath and are shown in fig. S14.

### Analyses of the cytokine level in mouse plasma samples

Immediately after euthanasia, blood samples were collected from mice in EDTA-treated tubes. The blood was centrifuged to separate the plasma, which was stored at −80°C. Twenty-five microliters of each plasma sample were diluted twice and then analyzed for the level of the various cytokines using the V-PLEX Proinflammatory Panel 1 Mouse Kit (MSD, cat. no. K15048D). The analysis was done on a MESO QuickPlex SQ 120 instrument according to the manufacturer’s instructions.

### Rigor and statistical analysis

Each key in vitro experiment was repeated at least twice. For statistical analysis, we used one-way analysis of variance (ANOVA) to compare different groups followed by Tukey’s post hoc test when the samples are normally distributed. Kruskal-Wallis (for unmatched groups) and Friedman tests (for matched groups) were used for statistical analyses of data, which were not normally distributed. Likewise, Student’s *t* test and Mann-Whitney *U* test were used for pairwise comparisons. Analysis was performed using GraphPad Prism 10. For animal experiments and on the basis of differences in means we consider to be biologically relevant and typical standard deviations seen in such data in previous studies, the number of animals needed for each group was estimated so that we can detect statistical significance at *P* < 0.05. We used only female mice as our studies are focusing on breast cancer and OC, which affect mainly female patients. Mice were assigned randomly to different groups. Laboratory personnel were not blinded during the animal experiments to avoid cross-contamination between groups. Each point on the graphs represents an individual mouse.
