# Supplementary material for: Leveraging dysregulated tumor metabolism for targeting anticancer bacteria
Source: Sci Adv. 2025 Jun 13;11(24):eads1630. doi: 10.1126/sciadv.ads1630 (PMC12164975; doi:10.1126/sciadv.ads1630)
Supplement: Supplementary file 1 — Figs. S1 to S15 Tables S1 to S3 [file sciadv.ads1630_sm.pdf]

Science Advances
AAAS

Supplementary Materials for

Leveraging dysregulated tumor metabolism for targeting anticancer bacteria

Akeem Santos *et al.*

Corresponding author: Ofer Reizes, reizeso@ccf.org; Mohammed Dwidar, dwidarm@ccf.org

Sci. Adv. **11**, eads1630 (2025)
DOI: 10.1126/sciadv.ads1630

This PDF file includes:

Figs. S1 to S15
Tables S1 to S3

Figure S1.

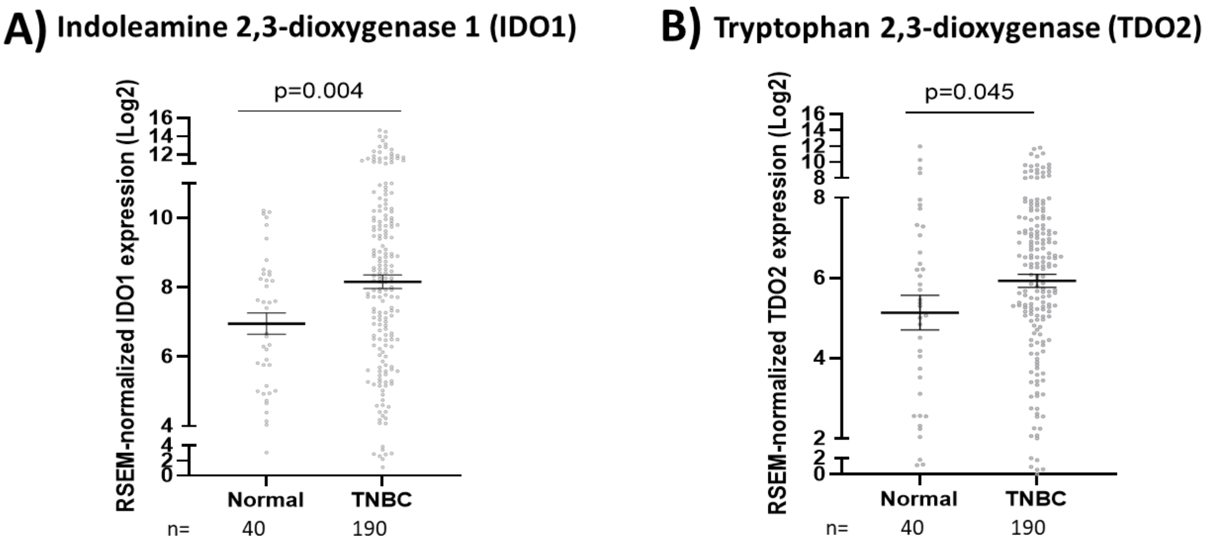

Figure S1: TCGA database analyses showing kynurenine pathway is upregulated in TNBC compared to healthy breast tissue specimens. A) IDO1 mRNA log2-normalized expression level is increased in TNBC compared to healthy controls in TCGA database. **B)** TDO2 expression levels are also increased in TNBC. lines show mean ±SE. Significance was tested using Mann-Whitney test.

Figure S2.

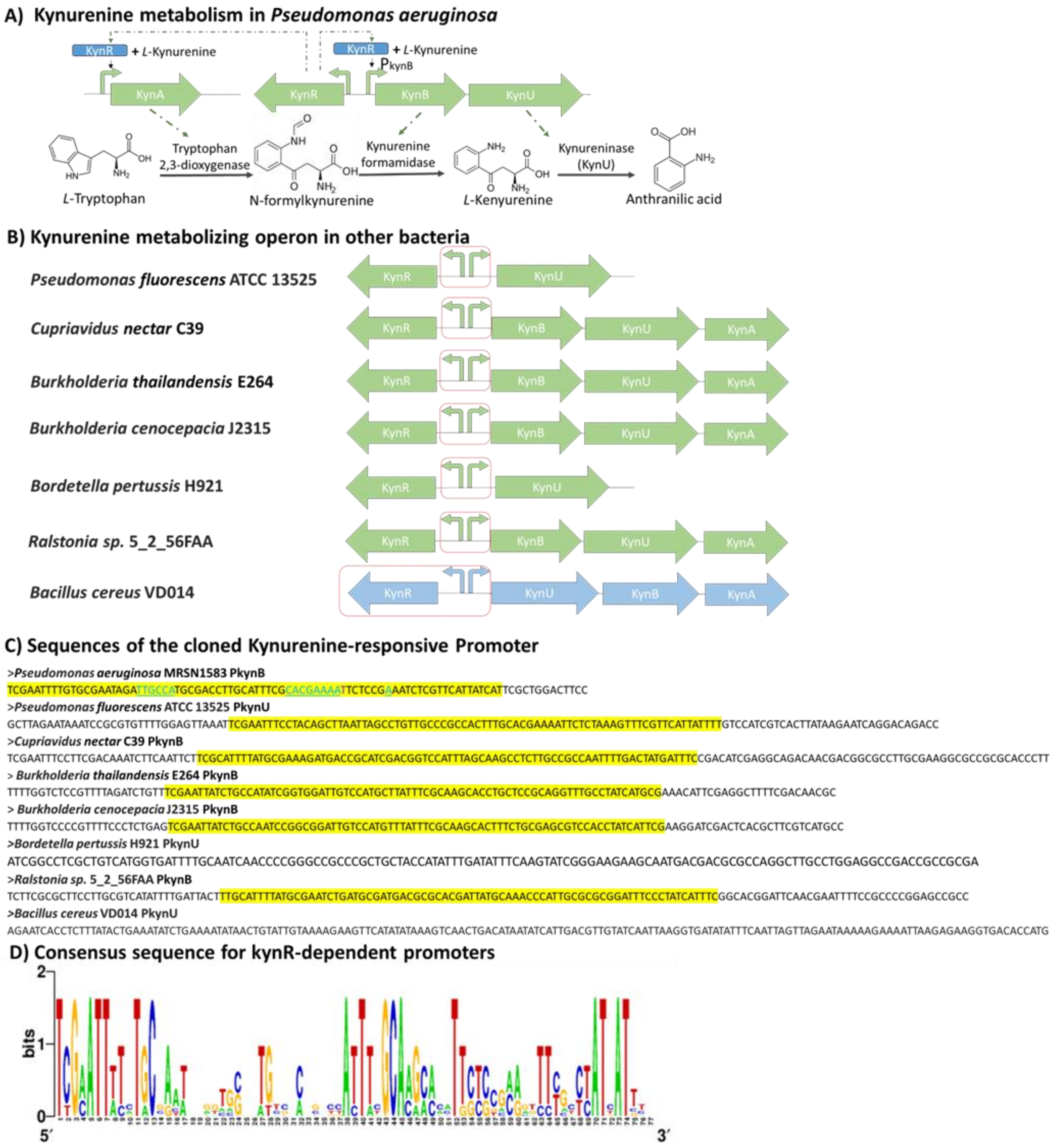

Figure S2: Sequences and gene neighborhood of the cloned kynurenine-responsive promoters. A) Kynurenine is sensed in *P. aeruginosa* as an intermediate metabolite in tryptophan to anthranilic acid metabolic pathway. Kynurenine binding to the transcriptional regulator KynR activates the transcription from the cognate P_{kynB} promoter.

B-C) Structure of the kynurenine metabolizing operons in selected Gram-negative and Gram-positive bacteria. The dotted red square shows the sequence in **(C)** which is cloned upstream of mCherry in **Figure** 2E to make plasmids pKynR-Pa-mCherry, pKynR-Cn-mCherry, pKynR-Pf-mCherry, pKynR-Bt-mCherry, pKynR-Bc-mCherry, pKynR-Bp-mCherry, pKynR-Rs-mCherry, and pCerKynR1-mCherry, respectively. All these plasmids harbored *P. aeruginosa* KynR except for pCerKynR1-mCherry. For pCerKynR1-mCherry, the original *B. cereus* kynR was incorporated in the plasmid rather than *P. aeruginosa* KynR since KynR in *B. cereus* belongs to tetR family regulators unlike KynR in Gram-negative strains, which is Lrp/AsnC family type regulator. **D)** Sequences highlighted in yellow in (**C**) were analyzed (https://weblogo.berkeley.edu/logo.cgi) to find the consensus sequence among the highly responsive Pkyn

promoters in Gram-negative bacteria. *B. pertussis* PkynB was excluded due to poor activity as shown in Figure 2E. *B. cereus* PkynU was also excluded. The sequence in blue in *P. aeruginosa* PkynB depicts the predicted -35, -10 and transcriptional start site based on the online BPROM promoter finding tool (http://www.softberry.com/berry.phtml?topic=bprom). For *P. aeruginosa* PkynB, there was originally an extra 5' sequence (not shown) which was trimmed in later experiments when found unnecessary for the activity.

Figure S3

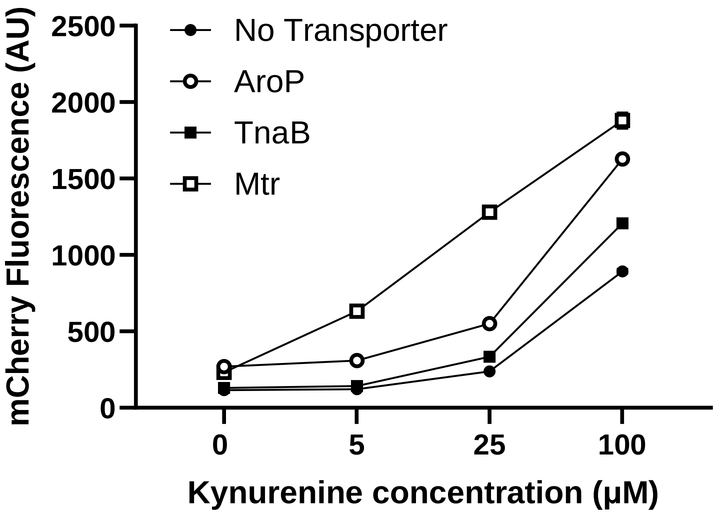

Figure S3: Comparing AroP, TnaB, and Mtr transporters for their ability to enhance the response to kynurenine in pPakynR2-mCherry plasmid backbone. The pPakynR2-mCherry plasmid together with the transporter-containing versions (pMtr-PakynR2-mCherry, pAro-PakynR2-mCherry and pTna-PakynR2-mCherry) were transformed in *E. coli*. *E. coli* harboring each of these plasmids were cultured in M9 media supplemented with 0.4% glucose +/- kynurenine at 5, 25, and 100 μM concentrations (n=3). mCherry fluorescence/OD_{600} were measured. Plotted are means ± SE.

Figure S4

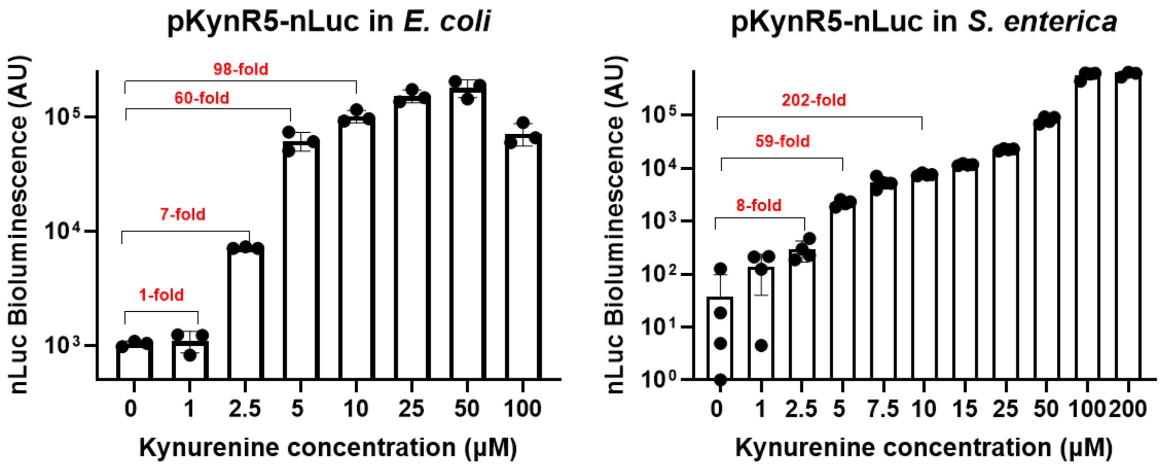

Figure S4: Testing pKynR5 dual plasmid system in *E. coli* and *S. enterica* using nLuc as a reporter protein. *E. coli* and *S. enterica* harboring pkynR5-nLuc dual plasmid system were cultured overnight in M9 media supplemented with 0.4% glucose +/- kynurenine added at the indicated concentration within 96-well plates and nLuc bioluminescence/OD_{600} was assayed after 24 h. Plotted are means ± SE. n=3.

Figure S5

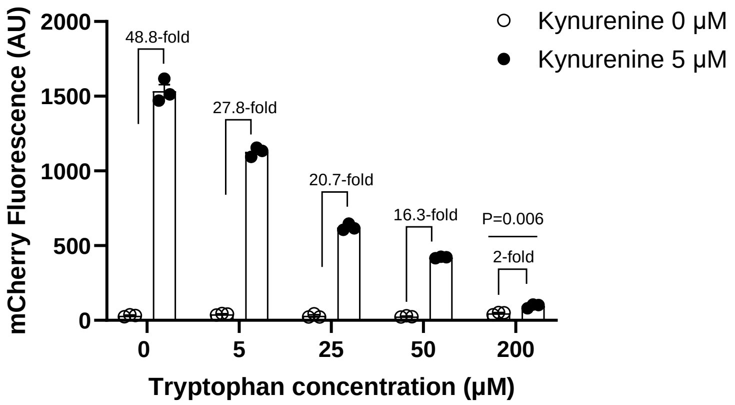

Figure S5: Effect of tryptophan on pKynR5 dual plasmid system performance. *E. coli* harboring pKynR5-mCherry dual plasmid system were cultured in M9 media supplemented with 0.4% glucose +/- kynurenine at 0 or 5 μM concentrations (n=3) in presence of tryptophan at the indicated concentrations within 96-well plates. mCherry fluorescence/OD_{600} was measured after 9 h. Plotted are means ± SE.

Fig. S6

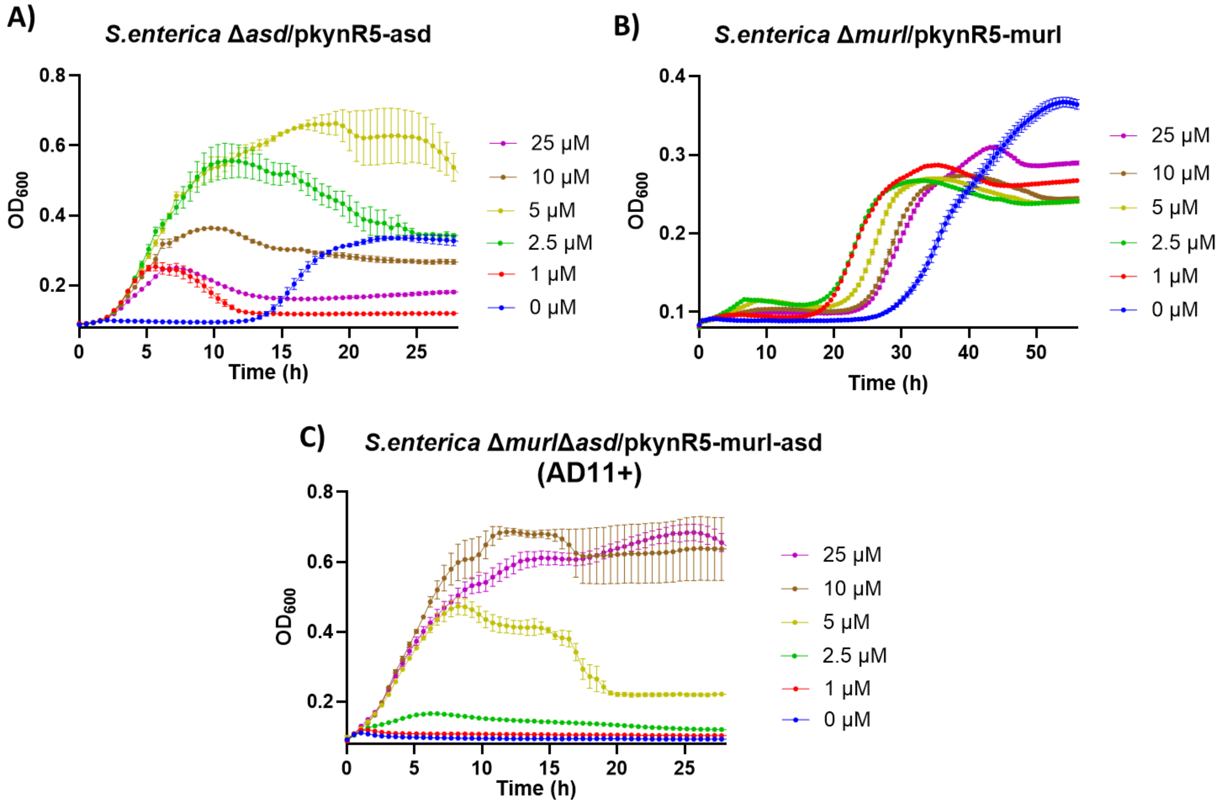

Figure S6: Attempts to control *S. enterica* growth in response to kynurenine by controlling *murI* and *asd* gene expression. A and B) Initial attempts aimed to make single gene knockouts in *S. enterica* (*asd* or *murI* gene) and supply this gene in pKynR5 dual plasmid system. **C)** Both genes were knocked out in the same mutant, and they were then supplied together in one operon on plasmid B of pkynR5 dual plasmid system. Growth of the mutants was assessed in M9 media at different kynurenine concentrations. M9 media was supplemented with glucose 0.4% and casamino acids 0.1%. Plotted are means ± SE of three replicates for each condition.

Figure S7

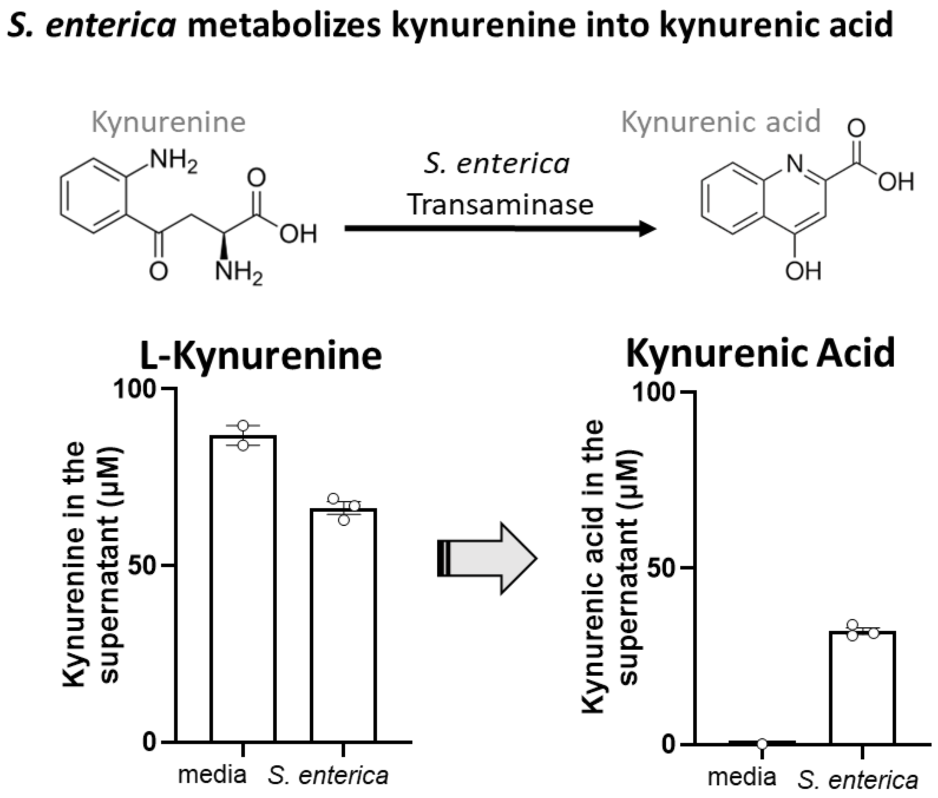

Figure S7: *S. enterica* metabolizes kynurenine into kynurenic acid. *S. enterica* ATCC 14028s was cultured overnight in M9 media supplemented with glucose 0.4% and kynurenine 100 µM. The cultures were then centrifuged. Spent media were filtered and analyzed for Kynurenine and kynurenic acid concentrations using LC-MS/MS.

Figure S8

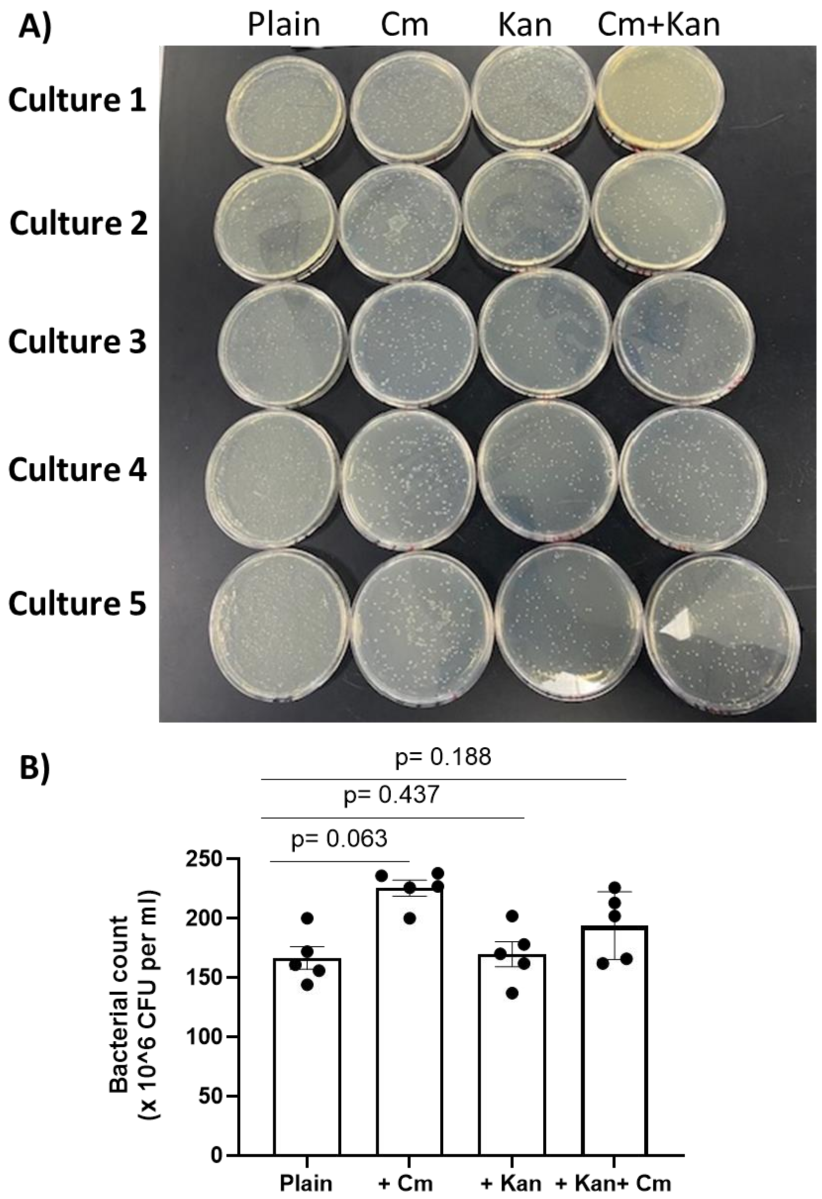

Figure S8: Both plasmids A, and B are stable in the engineered kynurenine-controlled *S. enterica* AD95+ strain. Plasmid A carries chloramphenicol-resistance gene cassette while Plasmid B carries kanamycin resistance gene cassette. *S. enterica* AD95+ was cultured overnight in LB broth supplemented with kynurenine at 20 µM without antibiotics, DAP or *d*-glutamate. Aliquots of these initial cultures were diluted 1:100 in fresh LB media supplemented with kynurenine only (without antibiotics, DAP or *d*-glutamate). Samples were then taken, centrifuged, and resuspended in PBS at OD 1.0 then plated on LB agar plates supplemented with *d*-glutamate and DAP with or without addition of antibiotics for colony counting. **A)** The results confirmed the stability of both plasmids as evidenced by similar number of colonies obtained for plain LB agar plates versus antibiotic-supplemented ones. **B)** On average OD 1.0 was equivalent to ~ 2 x 10^8 colony forming units (CFU)/ ml for all conditions.

Figure S9

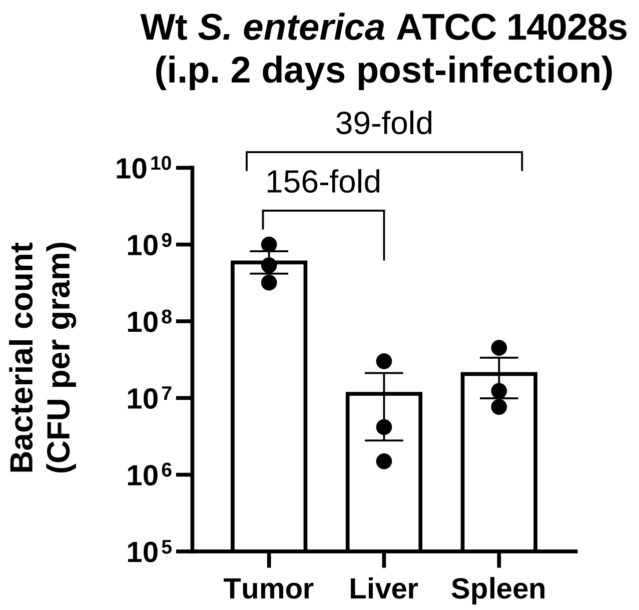

Figure S9: Wild-type *S. enterica* ATCC 14028s accumulates at high number in the liver and spleen after intraperitoneal injection. Subcutaneous 4T1 tumors were injected in BALB/c mice. Once tumors reached an average size of ~ 500 mm^3, Wild-type *S. enterica* ATCC 14028s were i.p. injected at a dose of approximately 2×10^6 CFU. Mice were euthanized 2 days later, and organs were harvested for CFU counting on LB agar plates. Bars represent means ± SE (n=3). Initially, four mice were injected with the bacteria. However, one mouse died prior to the pre-determined endpoint.

Figure S10

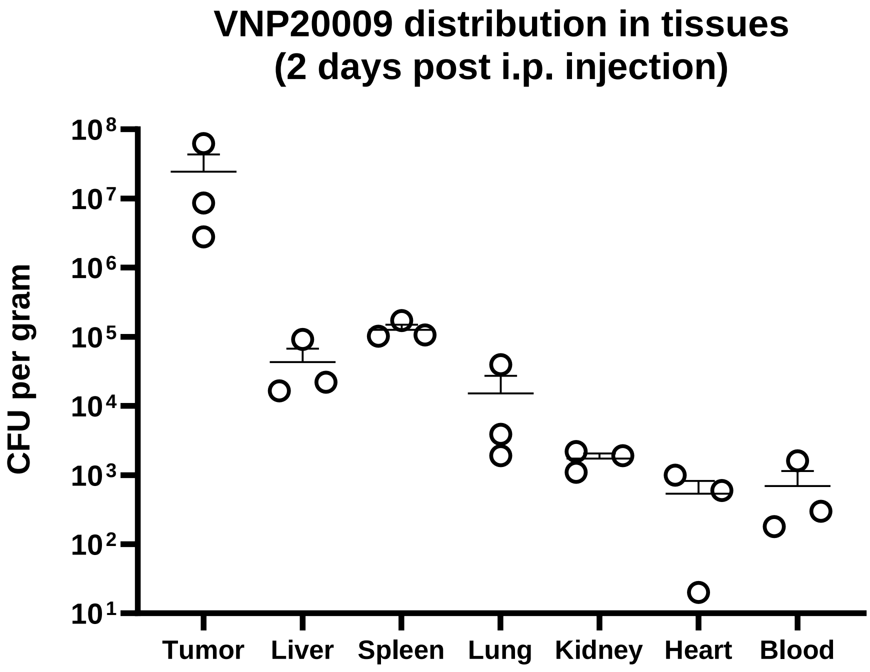

Figure S10: Parent VNP20009 strain accumulates in tumors at ratios 100~1000-fold higher than other organs after intraperitoneal injection. Subcutaneous KPCA.A tumors were injected in C57BL/6 mice. When tumors reached an average size of ~600 mm^3, *S. enterica* VNP20009 were i.p. injected at a dose of approximately 2×10^6 CFU. Mice were euthanized 2 days later, and organs were harvested for CFU counting on LB agar plates. Bars represent means ± SE.

Figure S11

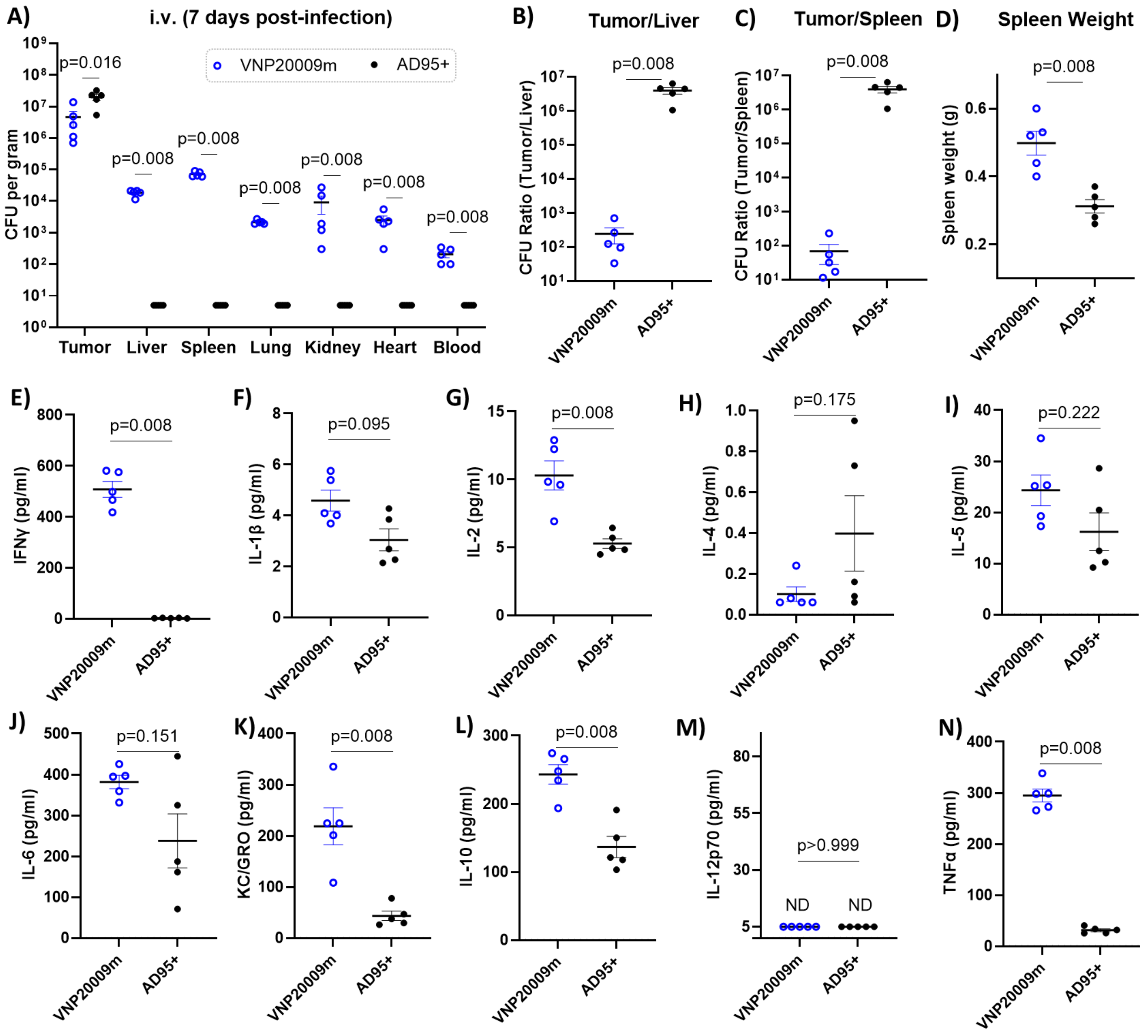

Figure S11. When injected intravenously, AD95+ has superior specificity to KPCA.A tumors compared to VNP20009m and results in significantly less splenomegaly and lower levels of plasma inflammatory cytokines. Subcutaneous KPCA.A tumors were injected in C57BL/6 mice. When tumors reached an average size of roughly 400 mm^3, *S. enterica* mutants were intravenously injected at a dose of ~ 2×10^6 CFU via retro-orbital injection. **A-C)** Mice were euthanized 7 days later, and organs harvested for CFU counting (n=5). Each symbol represents results from an individual mouse. Lines represent mean ± SE. When no colonies were detected at the highest dilution, the number was stated as 5 CFU/g tissue which is half the limit of detection. Statistical analysis was done by Mann Whitney test. p-values are presented in the panels. **D)** Spleen weights at endpoint. **E-N)** Blood was harvested from both groups at endpoint, processed for plasma, and analyzed for levels of inflammatory cytokines. IL-4 and IL-12p70 were detected in some but not all samples in our assay. When not detected, the values were stated at 0.06 pg/ml and 4.98 pg/ml which are corresponding to half the lower limit of detection for each, respectively.

Figure S12

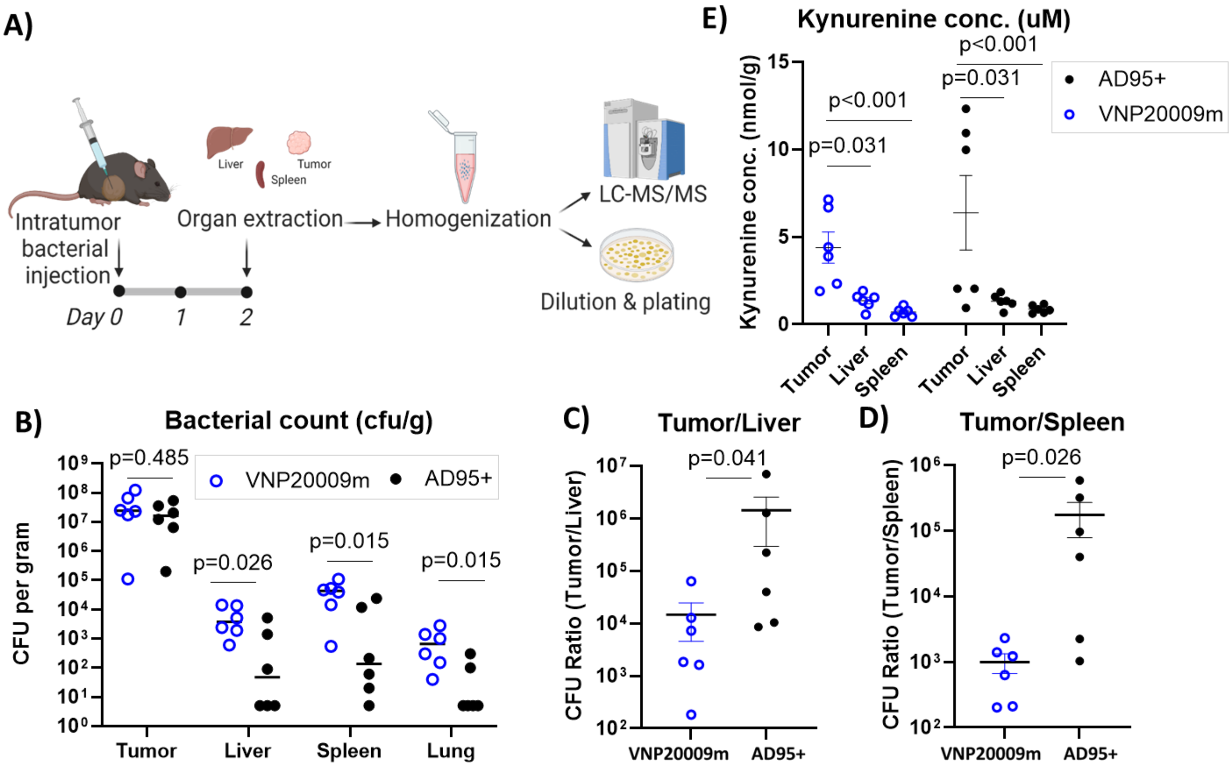

Figure S12. AD95+ accumulates at higher levels in tumors compared to VNP20009m when injected directly in subcutaneous KPCA.A tumors. A) Subcutaneous KPCA.A tumors were injected in C57BL/6 mice. When tumors reached an average size of ~ 600 mm^3, *S. enterica* mutants were intratumorally injected at a dose of approximately 2~4×10^6 CFU. Mice were euthanized 2 days later, and organs were harvested for CFU counting and measuring kynurenine concentration through LC-MS/MS. **B, C, D)** CFU counts in tumor versus other organs for each strain. **E)** Kynurenine concentration in the tumor versus liver and spleen, respectively. The experiment was repeated on different days, with n=2~3 for each group. Graphs depict cumulative data, with each symbol representing results from an individual mouse. Bars represent means ± SE. Statistical significance was determined by Mann Whitney test. When no colonies were detected on the agar plates at the highest dilution, the number was stated as 5 CFU/g tissue which is half the limit of detection (10 CFU/g tissue).

Figure S13

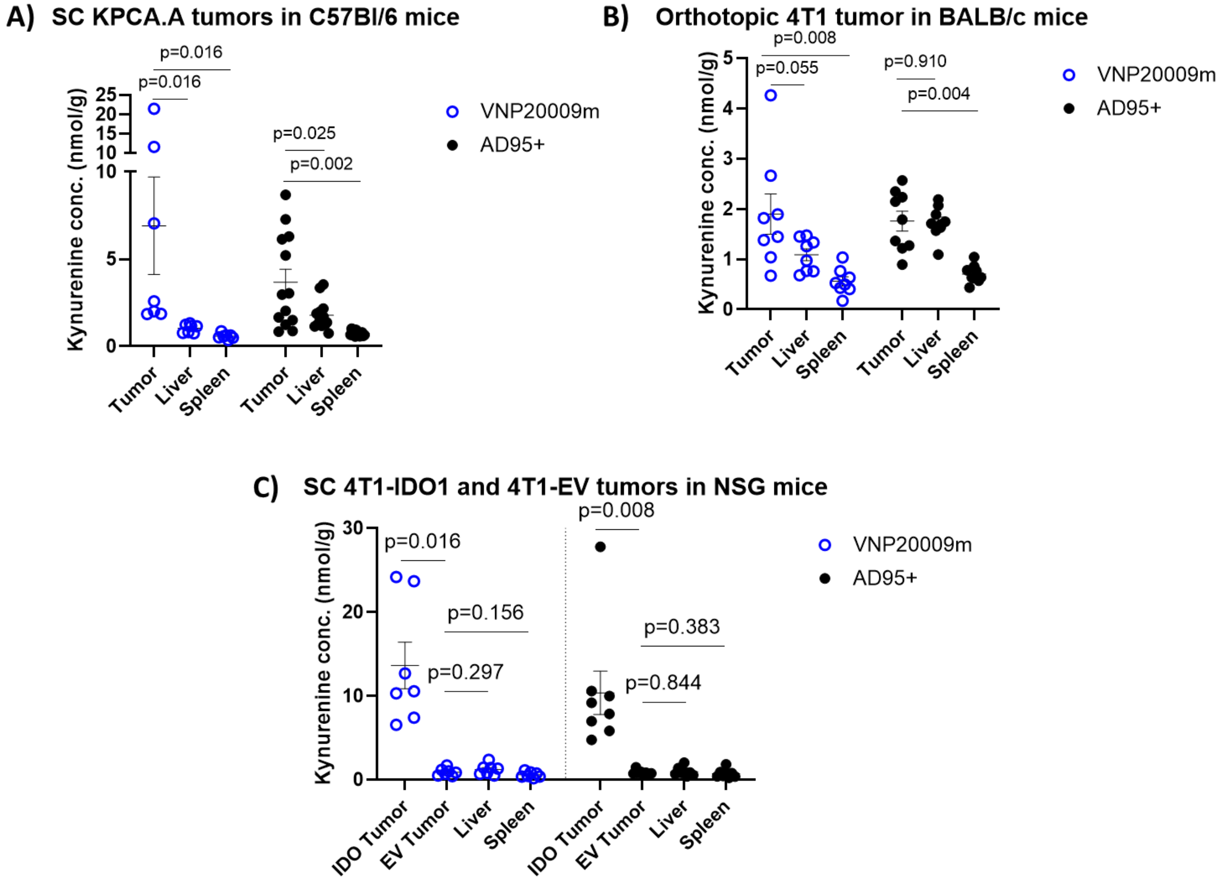

Figure S13: kynurenine concentration in tumors compared to spleen and liver for experiments in Figures 5 and 6. The LC-MS-MS analyses was performed on tissues harvested 2 days after i.p. bacterial injection. Points represent individual mice (n=6~8 for each group at each experiment). Bars represent mean ± SE. **A)** Subcutaneous KPCA.A tumors were injected in C57BL/6 mice. **B)** 4T1 breast tumors were injected in the 4th mammary fat pad in BALB/c mice. **C)** Subcutaneous tumors were injected in NSG mice on opposite flanks using both 4T1-IDO1 and control 4T1-EV cell lines. Statistical significance was determined by Wilcoxon test.

Figure S14.

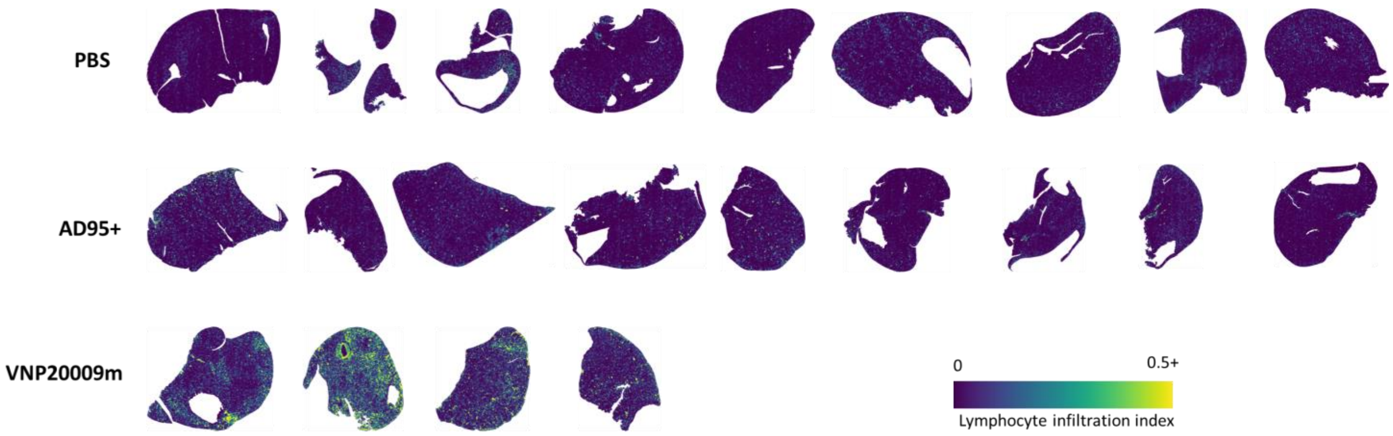

Figure S14: Heatmaps represent lymphocyte infiltration prediction score associated with the degree and distribution of lymphocytes in H&E-stained liver sections. These heatmaps were used to calculate the H-score for each specimen as shown in **Figure** 7D.

Figure S15.

A) Plasmids used in Figure 2A-F.

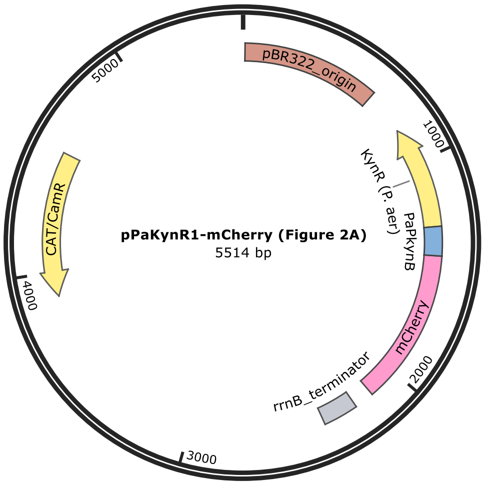

pPaKynR1-mCherry (Figure 2A)

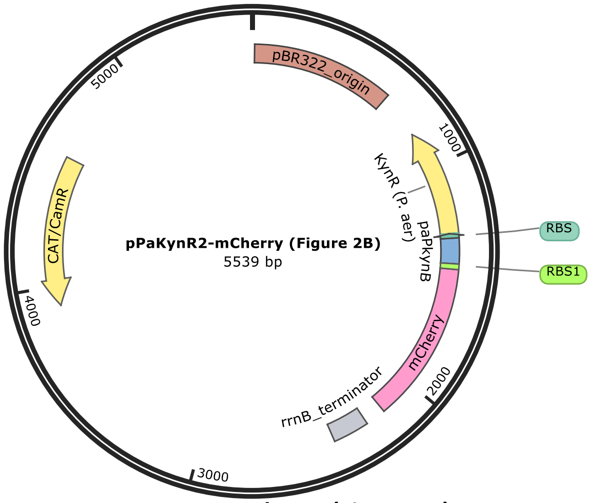

pPaKynR2-mCherry (Figure 2B)

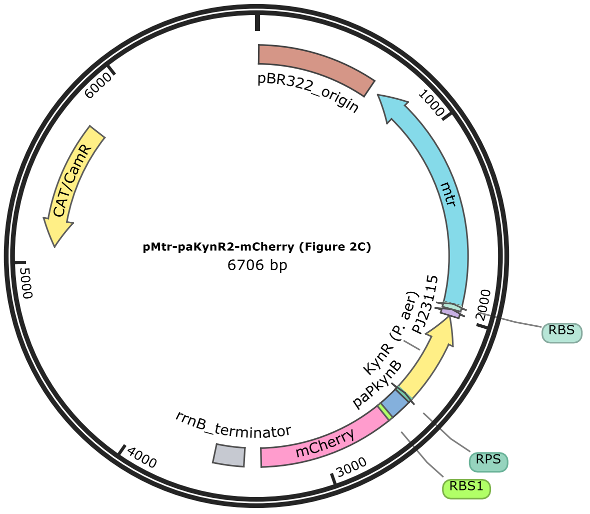

pMtr-PaKynR2-mCherry (Figure 2C)

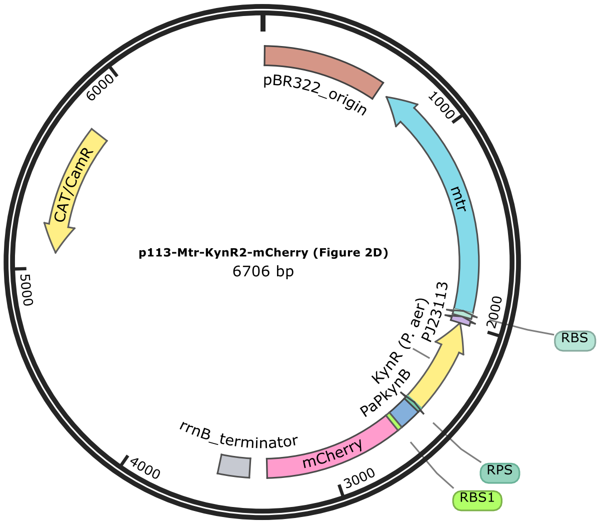

p113Mtr-PaKynR2-mCherry (Figure 2D)

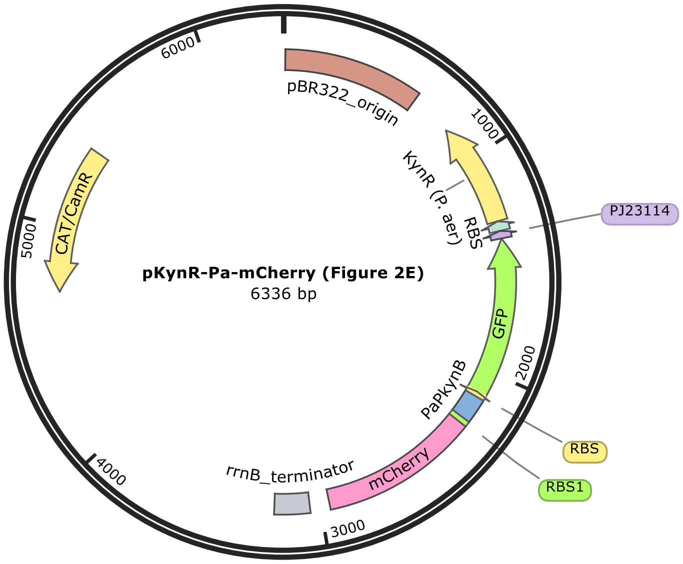

pKynR-Pa-mCherry (Figure 2E)

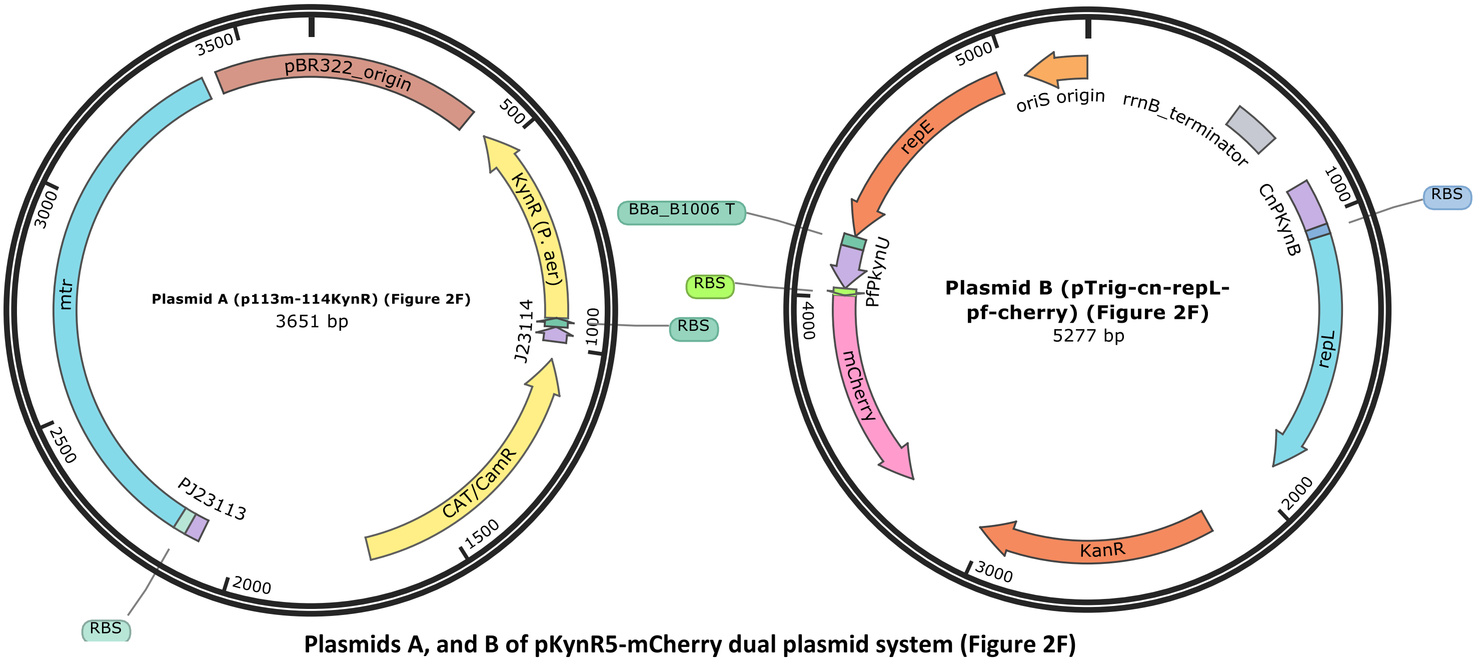

Plasmids A, and B of pKynR5-mCherry dual plasmid system (Figure 2F)

B) Plasmids used in Figure S6.

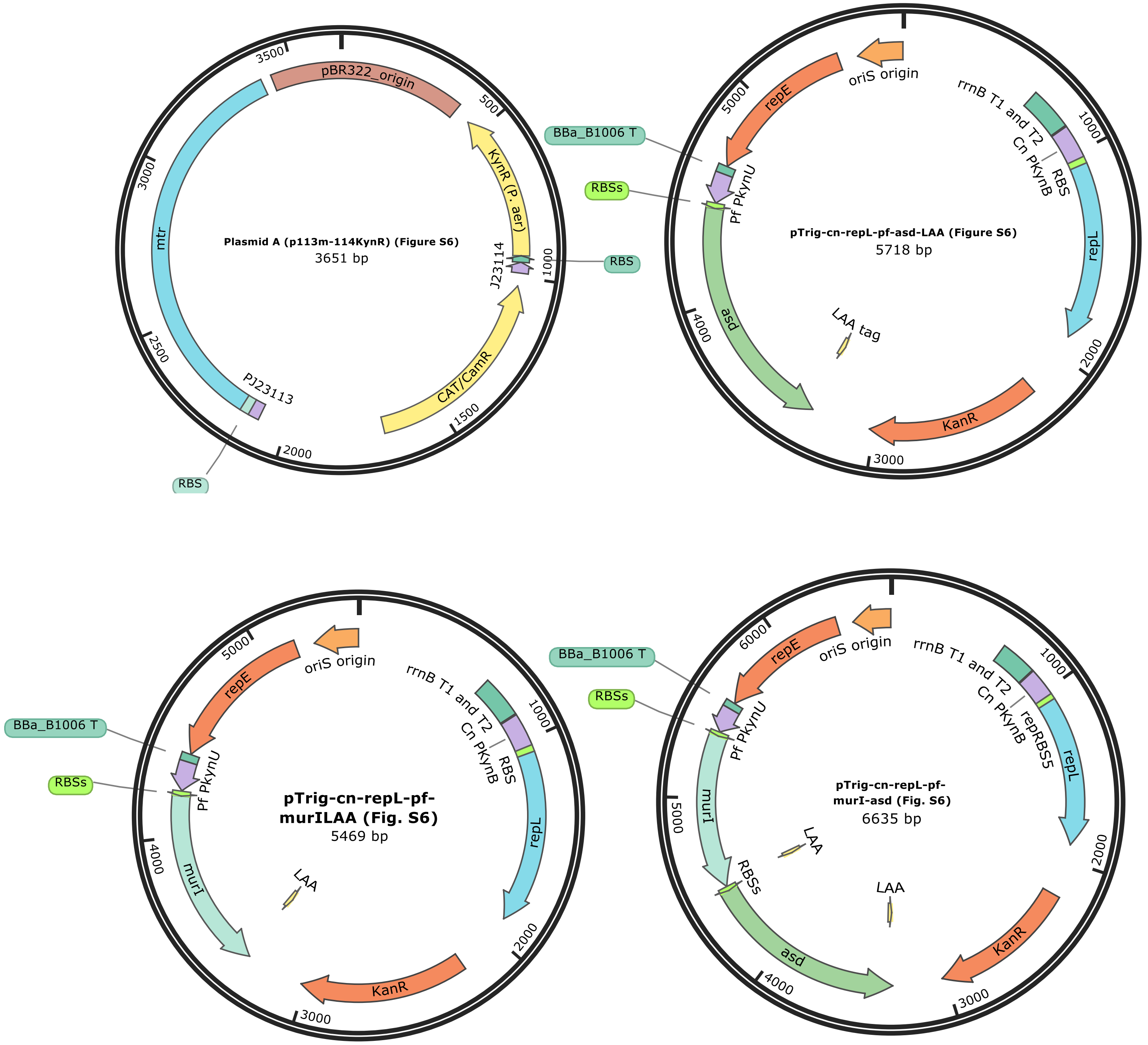

C) **Plasmids used in the modified pKynR7 system in Figure 4.**

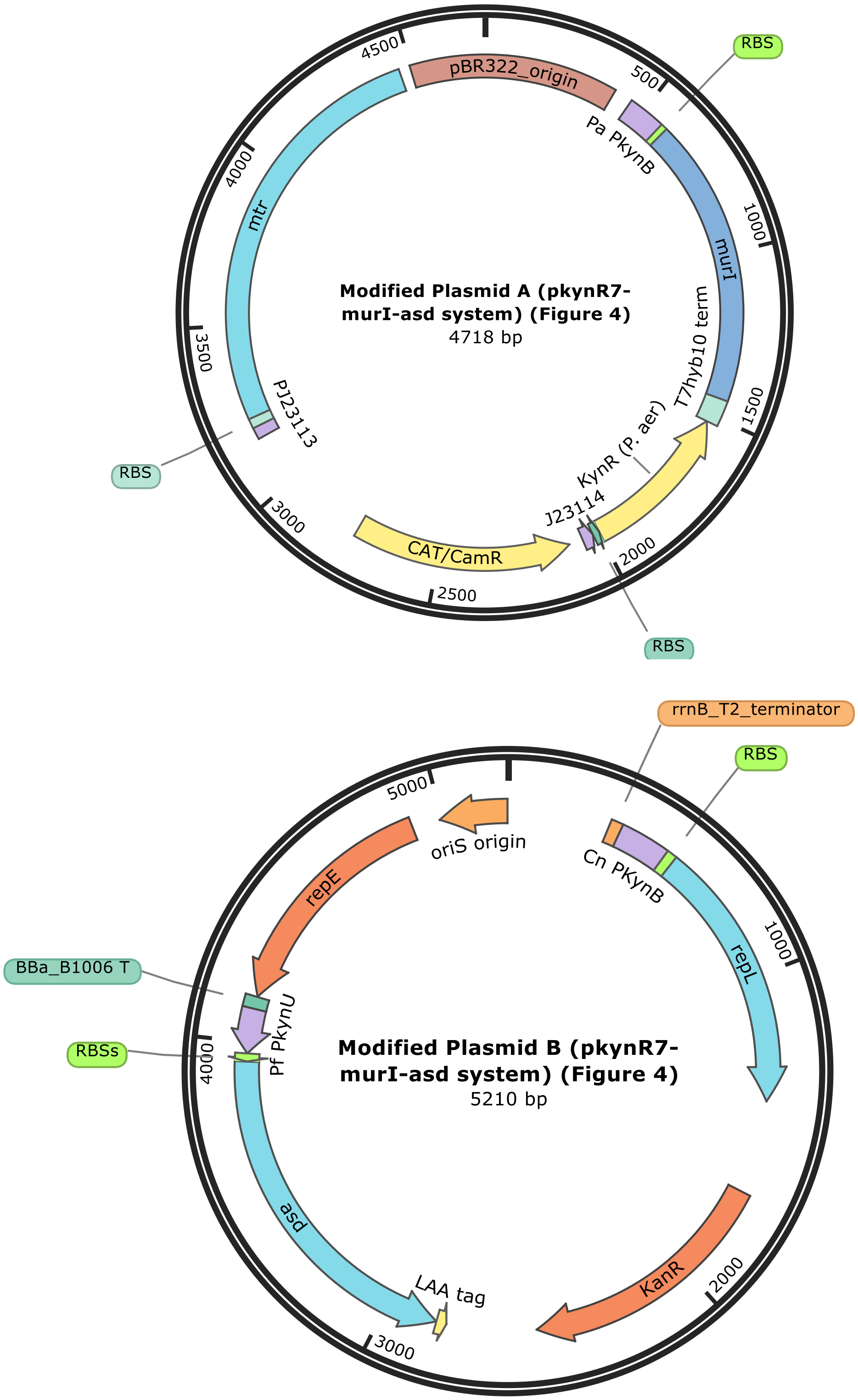

Figure S15. Maps of the key plasmids constructed during the course of the study. Plasmid maps were created using SnapGene Viewer (from Dotmatics; available at snapgene.com).

Table S1: Bacterial plasmids used in this study.

Plasmid	Features	Origin/ Antibiotic resistance	Figure
pPaKynR1-mCherry	$KynR_{P.aer.}$;<br>$P_{P.aer.KynB}$ – mCherry	pBR322 / Cm	2A
pPaKynR2-mCherry	$KynR_{P.aer.}$;<br>$P_{P.aer.KynB}$ – mCherry	pBR322 / Cm	2B, S3
pMtr-PaKynR2-mCherry	$KynR_{P.aer.}$;<br>$P_{P.aer.KynB}$ – mCherry;<br>P_{J23115} – Mtr	pBR322 / Cm	2C, S3
pTna-PaKynR2-mCherry	$KynR_{P.aer.}$;<br>$P_{P.aer.KynB}$ – mCherry;<br>P_{J23115}-TnaB	pBR322 / Cm	S3
pAro-PaKynR2-mCherry	$KynR_{P.aer.}$;<br>$P_{P.aer.KynB}$ – mCherry;<br>P_{J23115}-AroP	pBR322 / Cm	S3
P113-Mtr-KynR2-mCherry	$KynR_{P.aer.}$;<br>$P_{P.aer.KynB}$ – mCherry;<br>P_{J23113}-Mtr	pBR322 / Cm	2D
pKynR-Pa-mCherry	P_{J23114} – $KynR_{P.aer.}$;<br>$P_{P.aer.KynB}$ – mCherry;<br>GFP	pBR322 / Cm	2E
pKynR-Cn-mCherry	P_{J23114} – $KynR_{P.aer.}$;<br>$P_{C.nec.KynB}$ – mCherry;<br>GFP	pBR322 / Cm	2E
pKynR-Pf-mCherry	P_{J23114} – $KynR_{P.aer.}$;<br>$P_{P.flu.KynU}$ – mCherry;<br>GFP	pBR322 / Cm	2E
pKynR-Bt-mCherry	P_{J23114} – $KynR_{P.aer.}$;<br>$P_{B.tha.KynB}$ – mCherry;<br>GFP	pBR322 / Cm	2E
pKynR-Bc-mCherry	P_{J23114} – $KynR_{P.aer.}$;<br>$P_{B.cep.KynB}$ – mCherry;<br>GFP	pBR322 / Cm	2E
pKynR-Bp-mCherry	P_{J23114} – $KynR_{P.aer.}$;<br>$P_{B.per.KynU}$ – mCherry;<br>GFP	pBR322 / Cm	2E
pKynR-Rs-mCherry	P_{J23114} – $KynR_{P.aer.}$;<br>$P_{R.sp.KynB}$ – mCherry;<br>GFP	pBR322 / Cm	2E
pCerKynR1-mCherry	P_{J23114} – $KynR_{B.cer.}$;<br>$P_{B.cer.KynU}$ – mCherry;<br>GFP	pBR322 / Cm	2E
p113m-114KynR<br>(Plasmid A of pKynR5 system)	P_{J23114} – $KynR_{P.aer.}$;<br>P_{J23113} – Mtr	pBR322 / Cm	2F-H, S5
pTrig-cn-repL-pf-cherry<br>(Plasmid B of pKynR5-mCherry system)	$P_{C.nec.KynB}$ – repL;<br>$P_{P.flu.KynU}$ – mCherry	mini-F *ori* and P1 phage-derived *oriL* / Kan	2F-H, S5
pTrig-cn-repL-pf-nLuc<br>(Plasmid B of pKynR5-nLuc system)	$P_{C.nec.KynB}$ – repL;<br>$P_{P.flu.KynU}$ – nLuc	mini-F *ori* and P1 phage-derived *oriL* / Kan	S4
pTrig-cn-repL-pf-asdLAA<br>(Plasmid B of pKynR5-asd system)	$P_{C.nec.KynB}$ – repL;<br>$P_{P.flu.KynU}$ – asd_{LAA}	mini-F *ori* and P1 phage-derived *oriL* / Kan	S6
pTrig-cn-repL-pf-murILAA<br>(Plasmid B of pKynR5-murI system)	$P_{C.nec.KynB}$ – repL;<br>$P_{P.flu.KynU}$ – $murI_{LAA}$	mini-F *ori* and P1 phage-derived *oriL* / Kan	S6

pTrig-cn-repL-pf-murI-asd (Plasmid B of pKynR5-murI-asd system)	$P_{C.nec.\text{KynB}}$ – repL; $P_{P.flu.\text{KynU}}$ – murI_{LAA}-asd_{LAA}	mini-F *ori* and P1 phage-derived *oriL* / Kan	S6
p113m-114KynR-Pa-murILAA (Modified Plasmid A of pKynR7 system)	P_{J23114} – $\text{KynR}_{P.aer.}$; P_{J23113} – Mtr; $P_{P.aer.\text{KynB}}$ – murI_{LAA}	pBR322 / Cm	4-8, S11-S14
pTrig-cn-repL-pf-asdLAA (Modified Plasmid B of pKynR7 system)	$P_{C.nec.\text{KynB}}$ – repL; $P_{P.flu.\text{KynU}}$ – asd_{LAA}	mini-F *ori* and P1 phage-derived *oriL* / Kan	4-8, S11-S14
p101-Amp	–	Temperature sensitive pSC101 / Amp	–
p101-Amp-murI	P_{J23113} – murI	Temperature sensitive pSC101 / Amp	–
pMurI	P_{J23113} – murI	pBR322 / Cm	5-8, S11-S14
pKEx-Se-asd	*oriT*, *sacB*, Homologous arms for knocking out *asd*	pR6K / Kan	–
pKEx-Se-murI	*oriT*, *sacB*, Homologous arms for knocking out *murI*	pR6K / Kan	–

Table S2: Bacterial strains used in the study.

Bacterial strain	Features	Figure
Escherichia coli K12 (Steller, Takara)	Primary strain for cloning and testing genetic constructs	2A-G, S3-S5
Salmonella enterica ATCC 14028s	Parent *S. enterica* strain used to develop mutant AD95+	2H, 3, S4, S7, S9
S. enterica VNP20009	*S. enterica* ATCC 14028s Δ*purI*Δ*msbB*; ATCC BAA-3199 (YS1646)	S10
S. enterica VNP20009m	*S. enterica* VNP20009 Δ*murI/*pMurI	5-8, S11-S14
S. enterica Δ*asd*/pKynR5-asd	*S. enterica* ATCC 14028s Δ*asd*/pKynR5-asd	S6
S. enterica Δ*murI*/pKynR5-murI	*S. enterica* ATCC 14028s Δ*murI*/pKynR5-murI	S6
S. enterica AD11+	*S. enterica* ATCC 14028s Δ*murI*Δ*asd*/pKynR5-murI-asd	S6
S. enterica AD95+	*S. enterica* ATCC 14028s Δ*murI*Δ*asd*/pKynR7-murI-asd	4-8, S8, S11-S14

Table S3: Cancer cell lines used in the study.

Cell line	Features	Figure
4T1-luc	Murine triple negative breast cancer; Luciferase-expressing; BALB/c syngeneic.	1, 3, 6, S9, S13
4T1-EV	Derivative of 4T1-luc cell line through transfection with control (Empty) lentiviral vector.	3, 6, S13
4T1-IDO1	Derivative of 4T1-luc cell line through transfection with IDO1-expressing lentiviral vector.	3, 6, S13
KPCA.A	Genetically defined murine high grade serous ovarian cancer; C57BL/6 syngeneic.	1, 3, 5, 7, 8, S10-S14
BBPNM	Genetically defined murine high grade serous ovarian cancer; C57BL/6 syngeneic.	3
ID8	Murine high grade serous ovarian cancer; C57BL/6 syngeneic.	3
